# Co-design and feasibility of a pharmacist-led minor ailment service

**DOI:** 10.1186/s12913-021-06076-1

**Published:** 2021-01-22

**Authors:** Sarah Dineen-Griffin, Shalom I. Benrimoj, Kylie A. Williams, Victoria Garcia-Cardenas

**Affiliations:** 1 Charles Sturt University, Bathurst, Australia; 2 University of Granada, Granada, Spain; 3 University of Technology Sydney, Sydney, Australia

**Keywords:** Co-design, Minor ailment services, Qualitative research, Self-care, Community pharmacy, Community pharmacy services, Health services, Dissemination and implementation

## Abstract

**Background:**

Community pharmacies provide an appropriate setting to deliver minor ailment services (MASs). Many community pharmacy services have been developed previously without stakeholder involvement. As a result, implementation of services may fail to produce the expected impact. The aim of this research was to co-design and test the feasibility of an Australian MAS for minor ailment presentations.

**Methods:**

This study used co-design methodology which included two phases: (1) a focus group with stakeholders to allow the conceptualization of the service and agreement on service elements; (2) a literature review of clinical guidelines and three working meetings with a team of editors and general practitioners for the development of treatment pathways. Following this, a study evaluating the feasibility of the co-designed service was undertaken. The qualitative part of the methodology associated with the feasibility study comprised semi-structured interviews with MAS pharmacists, observation and completion of a tool by change facilitators identifying barriers and facilitators to service delivery. Qualitative data obtained for all phases were analysed using thematic analysis.

**Results:**

The developed service included the following components: (i) an in-pharmacy consultation between the patient and pharmacist, (ii) treatment pathways accessible to pharmacists on the internet to guide consultations, (iii) existing digital communication systems used by general practice to exchange patient information, (iv) training, and (v) change facilitation. As a result of feasibility testing, twenty-six implementation factors were identified for practice change, with the main change being the simplification of the pharmacist-patient consultation and data collection processes.

**Conclusions:**

An Australian MAS was generated as a result of co-design, while testing revealed that the co-designed service was feasible. As a result of integrating the views of multiple stakeholders, the designed MAS has been adapted to suit healthcare practices, which may increase the acceptance and impact of MAS when implemented into practice.

**Supplementary Information:**

The online version contains supplementary material available at 10.1186/s12913-021-06076-1.

## Background

International health systems are challenged with increasing rates of chronic illness and the associated clinical and economic burden represents a major barrier to the optimal provision of health care [1]. Evidence suggests that leveraging the potential of individuals to care for themselves (self-care or self-management) and involving them in decisions affecting their health is beneficial, particularly on the increasing rates of primary care consultations and ensuing health system pressures [2].

Self-care is highlighted by the World Health Organization (WHO) as integral to primary health care [3] and has the potential to make significant contributions to health system efficiency [4–6]. A policy statement released in 2019 by the International Pharmaceutical Federation and the Global Self-Care Federation, states the intention of the pharmacy profession and industry to further develop self-care as a “pillar of sustainable healthcare systems” [7, 8]. Self-care is usually the primary method for managing minor ailments [9]. Minor ailments are defined as “medical conditions that will resolve on their own and can be reasonably self-diagnosed and managed with non-prescription medicines” [10–13]. Conditions include strains and sprains, acute diarrhoea, constipation and the common cold, among others [14]. Self-medication is a fundamental component of self-care, and is defined by WHO as “the selection and use of medicines by individuals to treat self-recognized illnesses or symptoms” [15].

As health systems evolve to deliver “patient-centred” care, some key questions include: (1) how can primary health care professionals better support self-care and self-medication in an evidence-based, structured manner; and, (2) how can this be integrated into usual practice? Many health services have incorporated ways to increase patient involvement in their own health [16–18]. An example is that governments in Canada and the United Kingdom (UK) have been investing in supporting pharmacists to take on an expanded role in supporting self-care and responsible self-medication [19–21]. This was partially prompted by increases in general practice and emergency department (ED) presentations [21–25].

Minor ailment services (MASs) in the UK and prescribing for minor ailment (PPMA) services in Canada have been delivered by pharmacists since 2006 and 2007, respectively [26–29]. In the UK generally, MAS is open to all patients who are exempt from prescription charges and who are registered with a general practice in the local area. It allows individuals to consult a pharmacist for their minor ailment and receive treatment from the pharmacy at no cost [30]. PPMA was implemented in Alberta, Canada allowing pharmacists to prescribe medications for minor ailments. The remaining provinces have since adopted various degrees of prescriptive authority. Similar to MAS, patients consult with pharmacists for their conditions and pharmacists have the option of supplying medications from a limited formulary under PPMA [31].

These services were implemented as part of general health policy with various objectives including, improving accessibility to health care and relieving pressure on existing emergency services [32]. Spain [33], New Zealand [34] and Ireland [35] have evaluated or are currently evaluating the feasibility of similar initiatives. However, MAS requires contextualisation to the Australian health care system.

The implementation of new community pharmacy services into practice has been challenging and services often fail to produce the expected impact. This may be due to limited stakeholder involvement during service design, with many services previously developed intuitively without stakeholder involvement [36–38]. Co-design methodology is increasingly being applied for the development of pharmacy services [39–42]. The process engages service users, healthcare professionals and any other stakeholders with an interest in a particular service. As a result of co-design, services are adjusted to the specific health context in which they will be integrated, which in turn, increases their acceptability and feasibility in practice.

The aim of this research was to co-design and test the feasibility of an Australian community-pharmacy MAS aimed at enhancing self-care and responsible self-medication for patients presenting with minor ailments. The specific objectives of co-design were to:

(i) Generate a preliminary service model relevant to the Australian health system by identifying and agreeing on service elements; and
(ii) Develop treatment pathways for a selected number of minor ailments on an online platform (HealthPathways).

The specific objective of feasibility testing of the co-designed service was to:

(i) Explore the perceived barriers and facilitators to the provision of the service during pilot testing (eg. technology systems, documentation and data collection processes etc.) and examine why aspects of the service were/ were not feasible.

## Methods

The research adapted co-design methods previously applied by Sabater-Hernández et al. [39]. Sabater-Hernández used a three-step co-design process to develop a community pharmacy service including: (1) interviews and focus group with potential service users to identify key needs and concerns (2); a focus group with a group of stakeholders to generate a preliminary model of the service; and (3) focus group with community pharmacy owners and managers to explore the feasibility and appropriateness of the model [39].

**Fig. 1:**
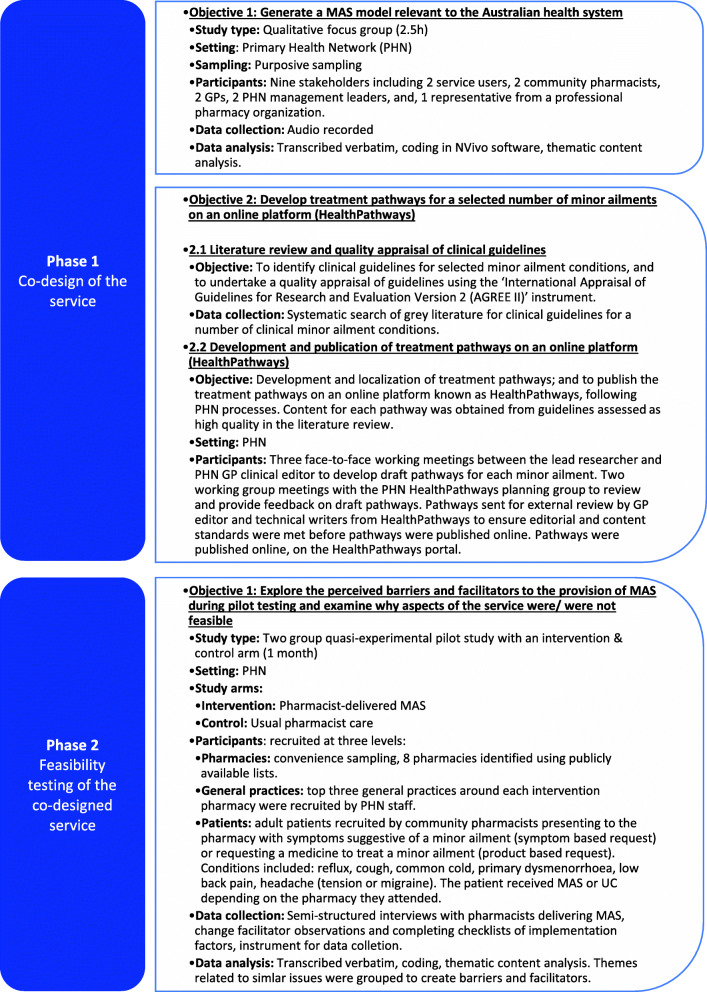
Study methodology

As such, the methodology in this study is similar however we have adapted each phase to meet the specific objectives of this research (Fig. 1).

### Phase 1: co-design of the service

#### Objective 1: generate a MAS model relevant to the Australian health system

The first phase engaged stakeholders in a 2.5-h qualitative focus group undertaken in June 2017, allowing researchers to conceptualize a minor ailments service model relevant to the Australian health system. Nine participants were purposively recruited and included: 2 service users, 2 community pharmacists, 2 general practitioners (GPs), 2 primary health network (PHN) management leaders, and 1 representative from a pharmacy organization. The GPs in the focus group were recruited directly by the PHN, given the research team was working with the PHN and within existing PHN governance.

A focus group guide was developed utilizing international literature pertaining to pharmacy care for minor ailments to guide discussion (additional file 1). This ascertained the elements of the model, investigated methods of integration and generated a list of potential barriers and facilitators to the delivery and implementation of MAS in Australia [26–29]. The focus group was moderated by an experienced qualitative researcher (SB). The moderator ensured only the neutral cues and prompts noted on the interview guide were used to limit the possibility of offering subjective opinion or critique. Responses were audio recorded, with consent, and transcribed verbatim by a transcription company. Data were managed in NVivo V.12 software (QSR International Pty; Victoria, Australia) [43].

Thematic analysis was used to analyze the data [44] which was conducted in accordance with Braun and Clarke’s (2006) framework, detailing six phases to thematic analysis [45]. The analysis was undertaken by one researcher (SDG) and discussed with investigators (VGC, KW, SB). Consensus was attained regarding themes (theme verification). The research was conducted in accordance with the consolidated criteria for reporting qualitative research (COREQ) [46]. The study received approval from the Human Research Ethics Committee at the University of Technology Sydney (reference: ETH17–1348). Participants provided written consent prior to participating in the focus group discussion.

#### Objective 2: develop treatment pathways for a selected number of minor ailments on an online platform (HealthPathways)

### 2.1 Literature review and quality appraisal of clinical guidelines

A literature review and quality assessment of clinical guidelines was undertaken for a number of minor ailments including reflux, primary dysmenorrhoea, headache (tension and migraine), common cold, cough, and low back pain. Ailments were selected on the basis they are able to be managed by self-care or self-medication.

A grey literature search was undertaken following methodology by Godin et al., described as “a feasible and robust method in applying systematic search strategies to identify resources in the grey literature” [47]. Initially, clinical guidelines of interest were identified by searching Google with a range of keywords and phrases. Search strategies were developed ensuring reproducibility of the guidelines of interest (additional file 2).

Guidelines for each ailment were included if they: (1) met the definition of a clinical guideline (“systematically developed statements which assist practitioner and patient decisions about appropriate healthcare for a clinical condition”) (2); proposed as a minimum, the assessment, management and referral points of the condition (3); intended for the management of adult patients (≥18 years); and (4) published or reviewed in Australia, Canada and the UK within the last 5 years. Older versions were excluded if more than one version of the same clinical guideline was found.

The first three hundred links from each grey literature search were imported in Microsoft Excel and saved without duplication for each condition. This was considered appropriate given it was a feasible number to screen while capturing relevant information [47]. Web links were first screened by title against eligibility criteria by one reviewer (SDG). All links identified in the search were saved and rechecked by a single reviewer (SDG). Full links were then assessed for eligibility by screening against inclusion criterion. Reference lists of included guidelines were reviewed to identify additional guidelines not retrieved in the original search. A second and third reviewer (VGC, SB) were consulted if a guideline was not able to be excluded with certainty. All authors (SDG, VGC, KW, SB) agreed on the guidelines for inclusion.

Guidelines included in the review were analyzed to identify components deemed relevant for the development of a treatment pathway. The guideline components were summarized in a pre-designed table in Microsoft Excel and included common signs/ symptoms, differential diagnosis, red flag symptoms requiring referral, pharmacological and non-pharmacological management. Data were extracted by one reviewer (SDG) and verified for accuracy by a second reviewer (VGC).

Following extraction, the quality of clinical guidelines were assessed using the ‘International Appraisal of Guidelines for Research and Evaluation Version 2 (AGREE II)’ instrument [48]. The AGREE II instrument comprises six domains and 23 items [48]. One reviewer (SDG) conducted the quality assessment for all guidelines, and two additional reviewers (VGC, SB) were consulted during this process. Any disagreements were resolved by discussion and consensus. Further detail on the quality assessment process is provided in additional file 2.

### 2.2 Development and publication of treatment pathways on an online platform (HealthPathways)

Each PHN in Australia establishes a clinical working group for the development of individual treatment pathways in their local area. These localized pathways are embedded on a web-based portal (known as HealthPathways), designed to be used at the point of care by health professionals [49]. The content in individual pathways is developed collaboratively by a GP clinical editor and a HealthPathways working group, following a process which translates clinical guidelines into HealthPathways [50]. Similarly, both clinical guidelines and treatment pathways are designed to aid health professional assessment and management of a particular condition. However, treatment pathways further provide specific guidance on management of a clinical condition, along with the aim of improving the coordination of care across disciplines and sectors (ie. detailing referral criteria, when and whom to refer to) [51].

The individual treatment pathways were developed for each clinical condition utilizing the results from the literature review outlined above. Following PHN processes [52], the lead researcher (SDG) worked with the PHN GP clinical editor to develop a draft pathway for each minor ailment condition during three face-to-face working meetings. This was followed by two working group meetings with the PHN HealthPathways planning group who provided feedback on the draft treatment pathways. Consensus was achieved following qualitative approval between researchers, the PHN GP clinical lead and the PHN HealthPathways planning group. Following the extensive development process, pathways were sent for review by external GP clinical leads and technical writers from HealthPathways, as part of the existing PHN process, to ensure editorial and content standards were met before being published online. Pathways were then published online on the HealthPathways portal, available for health professionals (including community pharmacists) to access in the PHN region of Western Sydney. A similar structure was followed for the development of each pathway (Table 1) [50].

**Table 1:** Structure of HealthPathways

Red flag referral criteria	Signs, symptoms or events recognized to be more serious in nature and point to the need for immediate referral for assessment.
Clinical assessment	Symptoms (duration, frequency and severity), past history of symptoms, medications used for this episode of symptoms or other health problems, known allergies and intolerances, other concomitant diseases or medicines.
Evaluation	Assessment of risk factors, contraindications and drug interactions.
Treatment	Evidence based nonpharmacological and pharmacological support recommendations.
Referral	Critical time of symptom evolution after which the pharmacist may suspect that it is not a minor ailment, as well as other symptoms or signs that point to the need for assessment by a general practitioner or another health provider, and the timeframe within which a patient is recommended to seek care.
Resources	Resources consulted in preparation of the pathway and patient resources to support self-care.

### Phase 2: feasibility testing of the co-designed service

#### Objective 1: explore the perceived barriers and facilitators to the provision of MAS during pilot testing and examine why aspects of the service were/ were not feasible

##### Description of feasibility study

This phase involved piloting MAS and determining the overall feasibility of the service elements (such as HealthPathways) in practice. The feasibility study used a two group quasi-experimental design. The study groups consisted of an intervention (pharmacist delivering the minor ailment service) and control (pharmacist delivering usual care) group. The duration of the trial was 1 month in October 2017. Eight pharmacies (5 intervention and 3 usual care) were purposively recruited in the PHN region of Western Sydney. These eight pharmacies ensured variability in the parameters of pharmacy size, type and location within the region. Pharmacy contact information was obtained from publicly available lists and pharmacies in the region were recruited via phone. Consent was sought at the pharmacy level from pharmacy owners, as per ethics approval. Intervention pharmacies identified three general practices around their pharmacies to be contacted by PHN staff to partake in the pilot trial. Consent was sought at the general practice level from practice managers. Consent indicated that GPs would receive health information from a pharmacist following consultation with a patient.

Patients were consecutively recruited by community pharmacies. Eligible patients were: (i) 18 years or over; (ii) requesting or self-selecting a medicine to treat symptoms (product-based presentation) and/or directly asking for pharmacists advice relating to their symptoms (symptom-based presentation) for one of the following minor ailments: reflux, cough, common cold, headache (tension or migraine), primary dysmenorrhoea, and back pain; (iii) attending the pharmacy in person; (iv) able to provide consent; and (v) contactable by telephone. Participants received the intervention or usual pharmacist care depending on allocation of the pharmacy to which the patient attended.

##### Intervention (minor ailment service)

Details of the intervention have been described in the TIDieR checklist (see additional file 3) [53] and in previously published peer-reviewed articles [54–56]. In brief, the minor ailment service included an in-pharmacy consultation between the patient and a pharmacist. The consultation was guided by the developed treatment pathways and communication systems which were developed and agreed during the initial co-design phase of the research. Pharmacists were trained for 7-h by researchers and GPs at Western Sydney PHN prior to delivery of the service and recruitment of patients into the study. As part of the intervention, pharmacies were provided 1-h weekly visits consisting of practice support and on-site training by a change facilitator (CF). The CF provides “support to help individuals and groups realize what they need to change and how to make those changes to incorporate evidence into practice” [57]. The role of CFs have been explored in pharmacy practice to guide and support the implementation of pharmacy services [58]. Further detail around the role of the CF is provided below.

##### Comparator (usual pharmacist care)

Pharmacists did not receive any of the intervention outlined above such as the support of a CF, clinical training and were not provided access to the treatment pathways. However, pharmacists did attend a 2-h workshop and were trained to use data collection software only.

##### Qualitative data collection

The qualitative methods adopted during the feasibility study included: (i) semi-structured interviews with intervention community pharmacists; and (ii) observations of pharmacists practice and completion of a facilitation tool by the CF detailing barriers and facilitators to intervention delivery. The specifics of each are as follows:

##### Semi-structured interviews with community pharmacists delivering MAS

Interviews with intervention pharmacists followed a semi-structured design and were based on research conducted in the UK examining stakeholder views of MAS delivery [26]. The aim of the interviews was to elicit qualitative feedback on community pharmacists’ views and experiences of a minor ailments service in Australia, and to determine the barriers and facilitators to service delivery (see additional file 4). Intervention community pharmacists were purposefully recruited via a phone call (SDG) given their participation in the feasibility study. Interviews were conducted by CFs (trained in qualitative research design) in a private and confidential area of the pharmacy, and were audio recorded.

##### Observation and completion of a tool by change facilitators detailing barriers and facilitators to MAS delivery

CFs undertook an analysis of implementation factors (additional file 5) following observation and discussion with the MAS pharmacist. CFs recorded data in a tool developed specifically for MAS and this research [59]. CFs were given a macro-enabled Microsoft Excel sheet (see additional file 6) with a number of parameters to record at each pharmacy visit including: (1) pharmacy name (2); visit number (3); the change barrier or facilitator identified (4); strategy chosen to overcome an identified barrier; and (5) resolution status. A barrier was defined as ‘any type of obstacle which may impede the delivery, implementation and/or sustainability of MAS’, while a facilitator was defined as ‘an element which can help to overcome barriers and/or accelerate the delivery or implementation of MAS’ [60]. As part of the change facilitation tool, CFs were provided a list of evidence-based change strategies from which they can choose one or more strategies to assist the intervention pharmacists or pharmacies in overcoming the change barrier. To increase credibility and reliability of CF findings, the CF participated in debriefing with the research team and engaged in reflection and re-examination of data at weekly intervals throughout the study period. Memos or notes detailing CF findings were also documented to ensure an audit trail.

Furthermore, intervention fidelity was monitored by CFs and the research team via the quality of data entered by pharmacists into data collection software. During visits, CFs asked intervention pharmacists about the practical application of web systems (e.g. HealthPathways) and ease of use of data collection software. This was to determine whether the systems were feasible for pharmacy practice, or if changes were required. CFs did not collect any data relating to the patient consultation.

##### Qualitative data analysis

Transcripts of interviews and data collected by CFs were verified by one investigator (SDG) while two investigators were responsible for the analysis of all qualitative data in the feasibility study (SDG, VGC). Data were managed in Microsoft Excel software. An inductive approach was used to develop a conceptual code structure for sorting of the data (following the process of thematic content analysis) [44]. Themes related to similar issues were further grouped to create one broad barrier or facilitator. The researchers ensured consistency in the coding of identified barriers and facilitators according to implementation factors identified from the Consolidated Framework for Implementation Research [61], the TICD checklist [62] and previous research on implementation factors related to pharmacy [63–65]. Implementation factors were organised into four different levels (patient, interpersonal, organisational, and system), as outlined by Hossain et al. [60]. The four levels were used as an overarching structure, for appropriate allocation and organisation of implementation factors identified.

The University of Technology Sydney Human Research Ethics Committee provided approval for the feasibility study and qualitative research (Reference: ETH17–1350). Participants were provided written study information. Consent was obtained for all participants at all levels.

## Results

### Phase 1: co-design of the service

#### Objective 1: generate a MAS model relevant to the Australian health system

Following the focus group, five components to the service model included: (1) In-pharmacy pharmacist-patient consultation, documentation of patient information and follow-up, (2) evidence-based treatment pathways on a web platform (HealthPathways), (3) communication channels between pharmacists and GPs on a web platform (HealthLink), (4) an educational training program, and (5) practice support. The results of the focus group are outlined below while the five service elements are described in detail in the TIDieR checklist (additional file 3).

#### In-pharmacy consultation, documentation and follow-up

Stakeholders proposed private consultations in the pharmacy (eg. in a consultation room) between the pharmacist and a patient when presenting with symptoms suggestive of a minor ailment or a medicine to self-treat their symptoms. Stakeholders acknowledged the value of the pharmacist-patient interaction in facilitating appropriate self-medication and referral when necessary. However, it was recommended that clear processes for referral be developed. Follow-up of patients was suggested to be incorporated following consultation with the pharmacist, to confirm whether patients sought care following a referral, or not (Q15 and 16; additional file 1). This was considered a priority by GPs in the focus group, as there was concerns that lack of follow-up of patients, particularly following referral to a medical professional, may delay diagnosis, treatment or lead to other lapses in safety. Stakeholders were initially doubtful of the potential to successfully follow-up patients in practice as most tend not return to the pharmacy given the potential self-limiting nature of their conditions (Q15; additional file 1).

Pharmacists in the focus group were initially doubtful of the service given the time demanding nature of their current positions and the time that would be required to document a consultation (Q3; additional file 1). Stakeholders considered self-care education as fundamental, however service-users noted the need for pharmacists to avoid medicalized language during the consult.

#### Technology platforms to promote collaboration

Technologies to facilitate collaborative practice were identified by stakeholders, including the use of existing GP software’s such as HealthPathways (Q13 and 14; additional file 1) and HealthLink (Q9 and Q10; additional file 1). Both pharmacists and GPs in the focus group felt that current communication is insufficient. GPs involved in the focus group were supportive of pharmacists using *HealthPathways* as a tool to facilitate their consultation and referral. GPs agreed that a structured referral process would be beneficial to ensure patients continually self-medicating without medical input were being identified and referred by the pharmacist. Moreover, they recognized that having support tools integrated with existing GP software’s would encourage pharmacists to use technologies in practice (Q11; additional file 1). Regular communication and an existing GP-pharmacist relationship was identified as important to maximise the success of MAS. It was suggested communication methods would need to be agreed with GPs to facilitate this relationship (Q9; additional file 1). Service users identified their privacy as important and sharing their health information with GPs should only be done following consent (Q12; additional file 1).

#### An educational training package and practice change support

It was agreed that pharmacists should receive refresher training to ensure competency in clinical areas, consultation skills, identifying red flag symptoms, and use of software. It was suggested that community pharmacists could be supported to deliver MAS by regular visits and telephone (Q2–5, additional file 1).

#### Objective 2: develop treatment pathways for a selected number of minor ailments on an online platform (HealthPathways)

The results of the literature review are summarised in PRISMA diagrams and the results of the quality assessment of clinical guidelines is summarized in additional file 7. The clinical guidelines identified as “high quality” were tailored to pharmacist’s scope in Australia following PHN processes, as previously mentioned. The seven treatment pathways are included in additional file 8.

### Phase 2: feasibility testing of the co-designed service

#### Objective 1: explore the perceived barriers and facilitators to the provision of the service during pilot testing and examine why aspects of the service were/ were not feasible

Nine semi-structured interviews were conducted with intervention pharmacists. Twenty site visits to intervention pharmacies were undertaken throughout the feasibility study, which included observation and completion of tool by CFs. Following analysis of both interview and CF data, twenty-six implementation factors were identified and organized into four levels (individual patient level (*n* = 4), interpersonal level (*n* = 14), organizational level (*n* = 7), and health system level (n = 1)). These factors were found to exist as a barrier, facilitator, or both (Table 2).

**Table 2:** Implementation factors influencing MAS delivery during feasibility testing

Level	Implementation factors	Description of implementation factors
Patient level	1.1 Pharmacist interaction with patient	The degree to which the pharmacist interacts with the patient.
Patient level	33. Customer needs	Real or perceived needs and demands of the patients.
Patient level	38 Patients’ awareness and observability	Patients’ background knowledge on the necessity of providing MAS through pharmacists, and their own need of receiving it.
Patient level	9. Observability	Level up to which the benefits of providing MAS are seen by individuals.
Interpersonal level	7. Complexity	Difficulty perceived for the implementation of MAS in the pharmacy, described by the duration, objectives and strategies required within the program.
Interpersonal level	12. Characteristics	Qualities, features or personalities of the providers and pharmacy owners, that will act as enablers or become barriers when providing MAS.
Interpersonal level	14. Individual stage of change	Stage at which each provider sits in relation to the evolution and progress over time.
Interpersonal level	15. Knowledge and experience	The extent to which the targeted individuals have skills, knowledge and experience that they need to adhere.
Interpersonal level	17. Auto efficacy	Provider’s self-beliefs to achieve the objectives established to provide and implement MAS.
Interpersonal level	19.1 Knowledge of own practice	The extent to which the targeted health care professionals are aware of their own practice in relation to recommended practice.
Interpersonal level	20. Team communication	Type, quantity, communication flow between the pharmacy’s staff around MAS.
Interpersonal level	23. Priority (relative) perception	Perception shared by the pharmacy’s workers about the importance of the implementation of MAS.
Interpersonal level	24. Culture	Expectations and shared values of all the pharmacy’s members.
Interpersonal level	28. Non-financial incentives	The extent to which individuals have non-financial incentives to adhere (e.g. personal recognition, CPD)
Interpersonal level	30. Internal supporters and opponents	Support provided by the pharmacy staff members for the implementation of MAS.
Interpersonal level	32. Leadership engagement	Commitment, involvement, capability and responsibility of the head of the pharmacy towards implementing MAS.
Interpersonal level	36 Relationship with physicians	Working relationships established between the pharmacy and its pharmacists and physicians within its surroundings.
Interpersonal level	37. Physicians’ awareness and observability	Perception and knowledge of physicians on the necessity of providing MAS through pharmacists.
Organizational level	2. Time	Amount of time devoted to providing MAS.
Organizational level	6. Resource use by staff	Level of use of the adequate bibliographical / technological resources to deliver MAS.
Organizational level	18. Teamwork	Abilities of the pharmacy’s staff to work together as a group.
Organizational level	19. Workflow (Team processes)	Way in which the pharmacy’s activities are divided and coordinated amongst its staff, including how pharmacy tasks are structured, how they are performed, in what order, how they are synchronised and how this affects the provision of service.
Organizational level	26. Structural characteristics	Pharmacy design, age, size and maturity in relation to the provision of MAS.
Organizational level	27. Resource availability	The extent to which the resources that are needed to adhere are available.
Organizational level	32.1 Capacity to plan change	The extent to which the targeted healthcare professionals have the capacity to plan necessary changes in order to adhere.
Organizational level/ Health system level	28.1 Financial incentives (service profitability)	The extent to which individuals have financial incentives or disincentives to adhere (e.g. ability to earn a profit from MAS).

#### Individual patient level

Factors at this level were related to individual patient needs, expectations, or previous experiences with community pharmacists and services. Pharmacists believed that time restraints of patients were a factor limiting receptibility to receive MAS and the expectation of the patient was also a barrier (Q1; additional file 4). Some pharmacists mentioned individuals selecting a medicine to self-treat their symptoms were less likely to engage in discussion with the pharmacist around their symptoms. Pharmacists expressed that normalizing MAS to the patient (ie. through advertising) may increase consumer receptibility to the service.

#### Interpersonal level

A valued aspect of MAS was the ability of pharmacists to directly engage with GPs (Q14; additional file 4). The majority recognized that establishing a means of communication and a relationship with GPs was important to improve information exchange. Pharmacists reported a variety of views with regard to the level of communication with GPs and indicated that the level of collaboration with GPs was variable as part MAS. Some pharmacists expressed that communicating with GPs had several barriers (eg. the patient did not have a regular GP, or there was no existing pharmacist-GP relationship prior to delivery of the service). Some pharmacists believed that it was helpful for the GP to be informed following each patient consultation, while others believed that only consultations resulting in referral should be relayed (Q13; additional file 4). Pharmacists commented that HealthPathways provided structure to consultation and the web platform was easy to navigate (Q8; additional file 4) and strongly indicated that the agreed pathways improved their confidence and knowledge to consult. Pharmacists identified that the training resources were relevant and necessary for MAS delivery. Multidisciplinary training was suggested as a way to improve collaboration (Q12; additional file 4).

#### Organizational level

Pharmacists agreed on the need for a private area in the pharmacy to conduct MAS, particularly if the service was to include more sensitive conditions such as vaginal thrush (Q6; additional file 4). This was viewed as difficult for some pharmacists where a private room was not available, particularly in smaller pharmacies. Others believed that a semi-private area was equally appropriate to maintain privacy for conditions of a less sensitive nature, such as cough or common cold. Most pharmacists suggested that lack of staff was a barrier to offering MAS (Q11; additional file 4). This was often related to the inability of the pharmacist to find time to deliver MAS as they were the sole pharmacist on duty, or did not have the support staff available to step away from the dispensary to consult. It was agreed the service must be provided by a pharmacist with an appropriate qualification and should not be delegated to a lesser-qualified staff member (Q3; additional file 4). All pharmacists commented on the importance of recording clinical encounters for accountability and follow-up. Simplified documentation processes and in-store training to assist with data collection, was recommended (Q10 and Q22; additional file 4).

#### Health system level

All pharmacists reported that remuneration for their time would be critical if they were to implement MAS in their pharmacy (Q15; additional file 4). Pharmacists had variable views towards reimbursement, however a fee between $10–30 (Australian dollars) per patient consult irrespective of medicine sale was deemed as appropriate (Q18; additional file 4). Pharmacists agreed that remuneration should not be associated with the sale of a medicine, since some consults may include only the provision of self-care advice (Q19; additional file 4). All but one pharmacist agreed that the government should provide this remuneration, with many suggesting that the economic savings with MAS implementation would cover remuneration (Q16; additional file 4).

## Discussion

The research details a co-design process that resulted in the development of a robust MAS aimed at encouraging self-care in Australia. This co-designed service was subsequently tested for feasibility. The qualitative data gathered during the initial focus group revealed the approach as an effective means of ascertaining stakeholder views and provided valuable input into MAS design. The input of pharmacists and GPs into the co-design process was important for understanding the practical application of MAS and existing systems allowing community pharmacists to better integrate with GPs.

### Comparison to international models

Ninety-four MASs (or PPMAs) have been identified internationally and implemented in regions across the UK and Canada [31]. While the literature pertaining to these MASs were studied to ensure structural characteristics of international services were considered during the design process [26–29], we did not duplicate these services. Rather, to ensure contextualisation and to focus the stakeholders during co-design, the research group presented a potential Australian model based on international literature and experience providing a starting point for the design of the service model. The service developed in this research presents both similarities (ie. consultation process) and distinct differences when compared to international MASs (ie. no implementation support by a CF is included as part of international MASs). Although some international initiatives require pharmacists to undertake training to deliver MASs, none have utilized a CF to assist with service implementation in practice [27].

### Previous challenges with implementation of international MASs

Nazar et al. noted the challenges with implementation of MASs and highlighted the importance of the design process for implementation success [66]. Multiple reasons have been identified including lack of GP engagement [26] and poor service design [66]. Aly et al. recommend involving GP stakeholders during the developmental process of MAS [26]. Views of stakeholders such as GPs, and policy makers, have been examined in the UK and Canada showing positive views on MAS being expressed [26, 67]. The co-design process in this research encouraged us to consider the feasibility of implementation and views of stakeholders throughout, as well as appropriateness to the Australian context.

Barriers and facilitators to the implementation of pharmacy services in Australia have been researched and reported from the perspective of community pharmacists [63–65]. This study, however, provides insight into the specific implementation factors affecting MAS delivery and implementation. The main facilitators to MAS were the developed treatment pathways, interprofessional collaboration between pharmacists and GPs, and the support and training by the CF. The main learnings from identifying these facilitators would be their use to enhance recruitment of patients and motivation of pharmacists in future implementation projects/ research. Barriers, such as limited time and patient acceptability were identified. To minimise the impact of these barriers for delivery and implementation, it is suggested that documentation and data collection processes are rationalized and restricted to primary outcome indicators only. Remuneration was described as essential for future MAS delivery. Remuneration is a continual theme in international pharmacy practice literature, with continual calls to ensure that community pharmacists are reimbursed for their time to deliver and document clinical services to patients [68–70]. As pharmacy teams start implementing services such as MAS, it is crucial to leverage the findings from this research to pre-empt possible barriers, tailor strategies according to their effectiveness, and improve implementation.

### Strengths and limitations

Several features strengthen our study. This study adapted previously published co-design methods by Sabater-Hernández et al., which led to the development of a pharmacy service aimed at patients with atrial fibrillation [39]. The approach was an effective means of ascertaining views of service users and health professionals. A limitation of our co-design approach, however, was the extent in the range and depth of engagement with participants, when considering the work by Arnstein [71, 72], Wilcox [73], Hart [74], and the NIHR guidance on patient involvement [75]. This, in turn, may have implications for comparability with interventions involving greater engagement with participants during co-design or where different approaches to co-design were applied altogether [76].

Additional limitations of the study included: (1) the qualitative work undertaken during feasibility testing only examined views of community pharmacists and did not consult other stakeholders due to resource limitations; and (2) while CFs were provided the same list of implementation barriers and facilitators and descriptions pertaining to both, CFs may have interpreted these differently. To overcome this limitation, CFs were trained by the research team prior to conducting pharmacy visits, and were asked to describe situations which led to their selection of a barrier or facilitator, to validate the data collected and strengthen study findings. Future research should aim to understand the views and experiences of MAS delivery by other stakeholders including GPs, patients, policy makers and organizations, facilitation strategies, and the impact of the CF. Further physician involvement in discussions are recommended to strengthen referral processes.

## Conclusion

An Australian MAS was generated as a result of this research. The research applied co-design methods for service design and was subsequently tested for feasibility in practice. The co-design method highlighted that stakeholders provide practical advice to service design and aided in the identification of systems used by medical professionals which, if also used by pharmacists, could promote integration of community pharmacy into broader primary care. This research contributes to the literature with a co-design approach involving stakeholders which may be applied for the development of other community pharmacy services. As a result of integrating the views of multiple stakeholders, the designed MAS has been adapted to suit healthcare practices, which may increase the acceptance and impact of MAS when implemented into practice.

## Supplementary Information

**Additional file 1. **Focus group discussion guide

**Additional file 2. **Search strategies for the literature review

**Additional file 3. **TIDieR checklist

**Additional file 4. **Semi structured interview guide for community pharmacists

**Additional file 5. **Implementation factors

**Additional file 6. **Example of completed facilitators database

**Additional file 7. **PRISMA diagrams and the results of the quality assessment process

**Additional file 8. **HealthPathways

## Data Availability

The datasets used and analysed during the study are available from the corresponding author on reasonable request.

## Abbreviations

AGREE II: International Appraisal of Guidelines for Research and Evaluation Version 2
CF: change facilitator
COREQ: consolidated criteria for reporting qualitative research
ED: emergency department
GP: general practitioner
MAS: minor ailment service
PHN: primary health network
PPMA: prescribing for minor ailment service
UK: United Kingdom
WHO: World Health Organization
