# Supplementary material for: Co-design and feasibility of a pharmacist-led minor ailment service
Source: BMC Health Serv Res. 2021 Jan 22;21:80. doi: 10.1186/s12913-021-06076-1 (PMC7821549; doi:10.1186/s12913-021-06076-1)
Supplement: Supplementary file 1 — Additional file 1. Focus group discussion guide [file 12913_2021_6076_MOESM1_ESM.pdf]

Additional file 1 Focus group discussion guide

Domain 1 Implementation and sustainability

1. How do you think the service would be best integrated into current practice in the beginning?
2. What are the facilitators to implementing the service?
3. What are the barriers to implementing the service?
4. What strategies would address the barriers to implementing the service you have identified?
5. What factors will ensure the sustainability of the service?

Domain 2 Collaboration with general practitioners

6. How should referrals between health providers take place (ie. pharmacists and GPs)?
7. What are your suggestions on method of referral (ie. letter given to patient)?
8. How should a relationship between the pharmacist and general practitioner be initiated?
9. How should communication between the pharmacist and general practitioner be performed?
10. When should communication between the pharmacist and general practitioner be performed?
11. What are your views on existing IT platforms for communication between community pharmacists and general practitioners?
12. What information do you think should be shared?

Domain 3 Treatment and referral pathways

13. What are your views on community pharmacists using agreed treatment protocols during consultation for assessment, management and referral for common minor ailments?
14. What are your views on existing eHealth platforms for pharmacists to access treatment protocols?

Domain 4 Documentation and follow up processes

15. Should follow up with patients be performed?
16. How and when should follow up with patients be performed?
17. Do you think should document their consultations? If so, what information should be documented?
