# Supplementary material for: Co-design and feasibility of a pharmacist-led minor ailment service
Source: BMC Health Serv Res. 2021 Jan 22;21:80. doi: 10.1186/s12913-021-06076-1 (PMC7821549; doi:10.1186/s12913-021-06076-1)
Supplement: Supplementary file 2 — Additional file 2. Search strategies for the literature review [file 12913_2021_6076_MOESM2_ESM.pdf]

Additional file 2 Search strategies for the literature review

Search strategy

The final search strategy used a combination of search terms as follows:

Headache	(headache OR "tension headache") AND (guideline OR "clinical guideline" OR protocol)
Migraine	(migraine) AND (guideline OR "clinical guideline" OR protocol)
Dysmenorrhea	(dysmenorrhea OR period OR menstrual OR menstruation) AND (pain) AND (guideline OR "clinical guideline" OR protocol)
Low Back Pain	(low back pain OR back pain) AND (guideline OR "clinical guideline" OR protocol)
Reflux	(GORD OR dyspepsia OR reflux) AND (guideline OR "clinical guideline" OR protocol)
Common cold	(common cold OR "upper respiratory tract infection" or cold or rhinovirus) AND (guideline OR "clinical guideline" OR protocol)
Cough	(cough) AND (guideline OR "clinical guideline" OR protocol)

The search was adapted to identify guidelines published in Australia, Canada and the United Kingdom where the search terms in combination with specific filters were used to limit the results, including site:au, site:ca and site:uk respectively.

Eligibility Criteria

For the purpose of this review, articles were initially included if they met the definition of a clinical guideline, written in the English language, intended for adult patients 18 years or older, the most current published version of the guideline and not a draft or summary. Guidelines were included if they were published for use in Australia, Canada or the United Kingdom. In addition, the guidelines were to be published or reviewed within the last 5 years.

Guideline Characteristics

Clinical guidelines identified to be included in the review were then analyzed to identify six key components deemed relevant in the development of a clinical pathway. The characteristic components included: common symptoms, diagnosis/assessment, red flags, pharmacological management, and non-pharmacological management. Guidelines were first assessed on whether the component was accounted for with each component being assessed as either present (+) or absent (-). Information from all included guidelines pertaining to each component were summarized in a pre-designed data extraction table.

Quality assessment

The AGREE (Appraisal Guidelines for Research and Evaluation) version II instrument is an internationally developed and tested appraisal tool, designed for the purpose of assessing the quality of clinical guidelines and considered the gold standard for guideline appraisal. The AGREE II instrument is comprised of six domains and two global rating systems with 23 items of interest to be assessed within clinical guidelines. The AGREE II domains include: scope and purpose; stakeholder involvement; rigour of development; clarity of presentations; applicability; and editorial independence. Twenty-three items make up the six domains and

are graded on a 7-point scale. The six domains are then scored as a total percentage of the maximum possible points they can score. The scaled domain scores are calculated as follows: (obtained score – minimum score)/ (maximum score – minimum score) x 100. To obtain the total score of each guideline, the above formula was utilized considering all six domains simultaneously. The AGREE II consortium has not set a fixed level to determine overall guideline quality but rather leaves the differentiating of high- and low-quality guidelines up to users. This is due to the use of the AGREE tool for use in different contexts and for different purposes as well as the relative importance of the six domains is expected to vary depending on the user's needs. Criteria based on previous guideline appraisals have been used to differentiate between low- and high-quality clinical guidelines. Therefore, a guideline was considered high quality and strongly recommended if the rigour and development domain and if an additional three or more domains in total scored ≥ 60%. Each clinical guideline included in the review was appraised using the AGREE II instrument (Table 1).

Table 1 AGREE II tool outline

**Domains**	**Assessment**
1. Scope and purpose	The overall objective, population and health question is clearly described.
2. Stakeholder involvement	The involvement of all relevant professional groups and target population has been sought out and clearly defined.
3. Rigor of development	Systematic literature search with grading evidence of the selected literature. Strength, limitations and methods for formulation of the recommendations are clearly described. Explicit link between evidence and recommendations. Externally reviewed by experts and has a procedure for updating.
4. Clarity of presentation	Provides specific and unambiguous recommendations. Different treatment options are clearly presented and key recommendations are easily identifiable.
5. Applicability	Describes facilitators and barrier to application as well as tools to implement. Resources in applying recommendations have been considered. Monitoring and audit criteria are present.
6. Editorial independence	Funding body has not influenced the content and competing interests of the developmental groups were addressed.
**Overall assessment**	**Overall quality and recommendations**
