# Supplementary material for: Co-design and feasibility of a pharmacist-led minor ailment service
Source: BMC Health Serv Res. 2021 Jan 22;21:80. doi: 10.1186/s12913-021-06076-1 (PMC7821549; doi:10.1186/s12913-021-06076-1)
Supplement: Supplementary file 3 — Additional file 3. TIDieR checklist [file 12913_2021_6076_MOESM3_ESM.pdf]

TIDieR
Template for Intervention Description and Replication

The TIDieR (Template for Intervention Description and Replication) Checklist*:

Information to include when describing an intervention and the location of the information

Number	Item	Where located **
1.	BRIEF NAME	Minor ailment service (MAS).
2.	WHY	MAS is a professional pharmacy service. A professional pharmacy service is defined as: "an action or set of actions undertaken in or organised by a pharmacy, delivered by a pharmacist or other health practitioner, who applies their specialised health knowledge personally or via an intermediary, with a patient/client, population or other health professional, to optimise the process of care, with the aim to improve health outcomes and the value of healthcare" (1). The international literature reports that MASs improve clinical, humanistic and economic outcomes for patients and the health care system (2, 3). At present there appears to be wide variability of practices on how pharmacist respond to patients presenting symptoms and to self-medication product request.
3.	WHAT	The MAS intervention was composed of:<br>1. Standardised consultation on an IT platform for pharmacist–patient intervention which included:<br>1.1. A consultation was provided by the pharmacist and followed collaboratively agreed protocols with general medical practitioners.<br>1.2. Each minor ailment studied had a collaboratively agreed protocol (see Procedures section).<br>1.3. Educational material for the patient included non-pharmacological treatment for each minor ailment.<br>1.4. An IT practice program led the pharmacists through the individual patient consultation and referral criteria for each minor ailment. The IT program guided pharmacists through two main pathways: patients presenting with symptoms or requesting a non-prescription medicine to self-treat.<br>2. Educational training for pharmacists: 7 hours of training was delivered by two experts (a community pharmacist and a general medical practitioner) which included: MAS procedure, clinical training, agreed service protocols, communication's skills with the patient and other health professionals, and data collection methods.<br>3. Change facilitators (CFs) made weekly on-site visits of 1 hour to support pharmacists and check fidelity of the intervention through data inspection. Support was also provided via email/telephone during the study period. The CFs were trained to ensure the study objectives were met. CFs were trained on a change facilitation approach that included: (1) The Generic Implementation Framework (GIF) which incorporates the stages of implementation and highlights the need to link factors, with strategies and evaluations for successful implementation (4); (2) Exploration of change barriers sourced from the Consolidated Framework of Implementation Research (CFIR), the Theoretical Domains Framework (TDF) and a previous implementation study in pharmacy practice; and (3) Change facilitation strategies.

**4.**	**PROCEDURES**	Intervention patients received MAS on presentation to the pharmacy. This involved a structured one-on-one face-to-face pharmacist-patient consultation with follow up. Pharmacists followed eight steps in the patient encounter:<br>1. Service offering, during which the pharmacist explains the features of the service;<br>2. Obtain written informed consent;<br>3. Establish the environment (i.e. take the patient to an appropriate or private consulting area);<br>4. Conduct an appropriate detailed assessment using HealthPathways (to confirm medications; medical conditions and allergies; identify symptoms; identify red flags or other referral criteria);<br>5. Use the agreed treatment protocols to determine if: (1) the patient would benefit from a non-prescription product; (2) the patient should be referred to a medical practitioner; (3) the patient only requires some verbal and written self-care advice. If a non-prescription product is recommended, the pharmacist will provide verbal and written self-care advice. If referral to another health care professional is indicated, the pharmacist will ensure this is done effectively by providing reasons for referral.<br>6. Using the IT platform (REDCap), completes all the data requirements at the time of the consultation;<br>7. Establish follow up plan (including how and when follow up will be conducted);<br>8. Notify the patients regular general practitioner using HealthLink (secure messaging system) when a medicine is provided or referral made;<br>The study involved a patient follow-up 14 days after the consultation. This follow-up was conducted by a member of the research team by telephone.<br><br>Specific pathways for each ailment are published in "HealthPathways Western Sydney". The pathways can be accessed via:<br>https://westernsydney.communityhealthpathways.org/. These protocols and guideline were codesigned and agreed between community pharmacists and general medical practitioners and include referral criteria according to patient's age, symptoms' duration, red flags, other health problems and special physiological situations such as pregnancy, breastfeeding; appropriate pharmacological and non-pharmacological treatment for each specific minor ailment.
**5.**	**WHO PROVIDED**	Registered pharmacists provided the MAS.<br>Pharmacy staff other than the pharmacists were not included in the study.
**6.**	**HOW**	MAS was provided through a face-to-face encounter between the pharmacist and the patient, so consultations were carried out in the community pharmacy. When patients attended the pharmacy either requesting a direct product request (non-prescription medicine) of presenting symptoms suggestive of a minor ailment they were informed about the study. If they accepted to participate the signed a consent form. 14 days following this consultation a researched phoned them at the number provided.

**7.**	**WHERE**	For the feasibility study, eight community pharmacies were purposively recruited in the region covered by the PHN of Western Sydney (5 pharmacies delivered the intervention (MAS) while 3 pharmacies delivered usual care (UC) and documented.
**8.**	**WHEN AND HOW MUCH**	The intervention was provided by the community pharmacist in a single consultation that took place when the patient visited the pharmacy asking for advice or requesting a non-prescription medication for one of the minor ailments included (see inclusion criteria in main text).<br>We asked intervention pharmacists to participate in:<br>-- 5-10 minute pharmacist-patient consultation;<br>-- 2 minutes to document each consultation;<br>-- 2 minutes to provide electronic feedback to the patient's usual GP per consultation (on patients consent);<br>-- 7 hours of training.<br>We asked intervention patients to participate in:<br>-- 5-10 minute pharmacist-patient consultation;<br>-- 2 minutes to complete the EuroQoL EQ-5D questionnaire;<br>-- 5 minute follow up telephone call with the research team.<br>(times were approximate and varied depending on the patient encounter)
**9.**	**TAILORING**	Eligible patients were recruited, if: (i) aged 18 years or over; (ii) requesting or self-selecting a medicine to treat symptoms (product-based presentation) and/or directly asking for pharmacists advice relating to their symptoms (symptom-based presentation) for one of the following minor ailments: reflux, cough, common cold, headache (tension or migraine), primary dysmenorrhoea, and back pain; (iii) attending the pharmacy in person; (iv) able to provide consent; and (v) contactable by telephone. Seven different protocols were used, one for each minor ailment. These conditions were identified as some of the most frequent minor ailments consulted in community pharmacy. These guidelines included referral criteria and treatment to be evaluated depending on patient's characteristics. The consultation followed two pathways depending on patients presenting with symptoms or requesting a medicine for a minor ailment. Those cases where the patient requested a medicine, the pharmacists had to evaluate if the medication requested was the most appropriate treatment.
**10.** ‡	**MODIFICATIONS**	No changes were made during the course of the study.
**11.**	**HOW WELL (planned)**	The MAS was codesigned with stakeholders. This included consumer representatives, community pharmacists, GPs, primary health network (PHN) management leaders, and a representative from a pharmacy organization. The GPs associated with the focus group were recruited directly by the PHN. The specific pharmacist/patient interventions were developed with this group and then piloted. A CF made monthly on-site visits in every community pharmacy to assess adherence to the treatment pathways. The CF completed a tool at each pharmacy including the collection of barriers to delivering the service. The CF checked the fidelity of the intervention through checks on the data

		collection program and the extracted data from this program. The CF was available telephone and email contacts to assist pharmacists in the intervention group throughout the study.
**12.‡**	**HOW WELL (actual)**	In addition to further assess intervention adherence and fidelity, the IT data collection program was checked by the research group. The consultation was documented though the IT practice program (REDCap). The pharmacist recorded his/her actions in the program. Evidence for those cases where community pharmacists didn´t adhere to the treatment pathways, for example, when the patient was not appropriately referred were recorded as part of the process.

References:

1. Moullin JC, Sabater-Hernández D, Fernandez-Llimos F, Benrimoj SI. Defining professional pharmacy services in community pharmacy. Res Social Adm Pharm. 2013;9(6):989-95.
2. Aly M, García-Cárdenas V, Williams K, Benrimoj SI. A review of international pharmacy-based minor ailment services and proposed service design model. Res Social Adm Pharm. 2018;14(11):989-98.
3. Dineen-Griffin S, Benrimoj SI, Rogers K, Williams KA, Garcia-Cardenas V. Cluster randomised controlled trial evaluating the clinical and humanistic impact of a pharmacist-led minor ailment service. BMJ Quality & Safety. 2020:bmjqs-2019-010608.
4. Moullin JC, Sabater-Hernández D, Fernandez-Llimos F, Benrimoj SI. A systematic review of implementation frameworks of innovations in healthcare and resulting generic implementation framework. Health Research Policy and Systems. 2015;13(1):16.
