# Supplementary material for: Co-design and feasibility of a pharmacist-led minor ailment service
Source: BMC Health Serv Res. 2021 Jan 22;21:80. doi: 10.1186/s12913-021-06076-1 (PMC7821549; doi:10.1186/s12913-021-06076-1)
Supplement: Supplementary file 4 — Additional file 4. Semi structured interview guide for community pharmacists [file 12913_2021_6076_MOESM4_ESM.pdf]

Additional file 4 Semi structured interview guide for community pharmacists

Domain 1 Acceptability and perceived benefits of the service

1. What are your views on minor ailments services?
   - For the pharmacy as a business?
   - For pharmacists as a healthcare professional?
   - For the patient?
   - For the healthcare system?
2. Do you believe that a minor ailment service is important? Why/ why not?
3. Do you think providing the service fits your role as a community pharmacist?
4. Do you think the service is appropriate for your pharmacy?
5. Do you think the service ensures the appropriate and safe provision of health care to patients?
   Why/ why not?

Domain 2 Service flow and elements

6. Do you think that the pharmacy offers adequate privacy to offer consultations for minor ailments? Can you suggest any improvements?
7. Have you adapted or modified the service or workflow in any way?
8. What are your views on the treatment protocols (HealthPathways)? Do they effectively support you?
9. What are your views on the referral points and processes? Are these adequate?
10. What are your views on the documentation and record keeping process?

Domain 3 Resources

11. Do you think there is enough resources (eg. time, space, staff) to deliver the service in this pharmacy?

Domain 4 Training and support

12. Do you believe the training and ongoing monthly in-store and telephone support is adequate to support you to deliver the service? Why/ why not?

Domain 5 Interprofessional collaboration

13. When an individual patient consults at the pharmacy, do you believe it is valuable for their GP to be informed or notified by an official channel (ie. HealthLink)? Why/ why not?
14. What are your views on the level of healthcare professional collaboration- is this adequate? Should there be more/ or less?

Domain 6 Remuneration

15. Do you believe pharmacies should be remunerated to provide the service in Australia?
16. Who should fund the service?
    a. Patient?
    b. Government?
    c. PHN?
17. Do you think there should be a cost associated with patients accessing the service?

18. What do you believe is an appropriate level of reimbursement?
19. Do you think the service would change if there was no monetary incentive other than associated sale revenue?

Domain 7 Implementation and sustainability

20. Are there any barriers or facilitators associated with your offering of the service in your pharmacy? Please describe (if any).
21. Do you believe that there are any factors (internal or external) that would influence your decision to implement the service?
22. What aspects of the service do you think could be improved?
23. Are there any aspects of the service that do/do not work well that haven't been covered?
24. Do you have any other views or experiences regarding the service?
