# Supplementary material for: Co-design and feasibility of a pharmacist-led minor ailment service
Source: BMC Health Serv Res. 2021 Jan 22;21:80. doi: 10.1186/s12913-021-06076-1 (PMC7821549; doi:10.1186/s12913-021-06076-1)
Supplement: Supplementary file 5 — Additional file 5. Implementation factors [file 12913_2021_6076_MOESM5_ESM.pdf]

Additional file 5 Implementation factors

No.	Implementation factor	Description of Implementation Factor
**1**	Communication with patients	Pharmacist's skills when communicating with patients during MAS.
**1.1**	Pharmacist interaction with patient	The degree to which the pharmacist interacts with the patient with the aim of 80% pharmacist forward orientation.
**2**	Time	Amount of time devoted to providing MAS.
**6**	Resource use by staff[4]	Level of use of the adequate bibliographical / technological resources to deliver MAS.
**7**	Complexity[1]	Difficulty perceived for the implementation of MAS in the pharmacy, described by the duration, objectives and strategies required within the program.
**9**	Observability[2]	Level up to which the benefits of providing MAS are seen by individuals.
**10**	Compatibility (individual alignment)[1]	The degree of tangible fit between meaning and values attached to the intervention by involved individuals, how those align with individuals' own norms, values and perceived risks and needs.
**11**	Awareness of the change[2]	The extent to which the individuals in the pharmacy are aware of and familiar with the recommendations.
**12**	Characteristics[1]	Qualities, features or personalities of the providers and pharmacy owners, that will act as enablers or become barriers when providing MAS.
**14**	Individual stage of change[1]	Stage at which each provider sits in relation to the evolution and progress over time.
**14.1**	Emotions[2]	The extent to which emotions affect adherence e.g. enthusiasm, frustration, cognitive overload, tiredness, regret.
**14.2**	Individual identification[1]	How individuals perceive the organisation and their relationship, job satisfaction, degree of commitment with it.
**15**	Knowledge/ experience[3]	The extent to which the targeted individuals have skills, knowledge and experience that they need to adhere.
**17**	Auto efficacy[1,2]	Provider's self-beliefs to achieve the objectives established to provide and implement MAS.
**18**	Teamwork[4]	Abilities of the pharmacy's staff to work together as a group.

**19**	Workflow (Team processes)[2]	Way in which the pharmacy's activities are divided and coordinated amongst its staff, including how pharmacy tasks are structured, how they are performed, in what order, how they are synchronised and how this affects the provision of service.
**19.1**	Knowledge of own practice [2]	The extent to which the targeted health care professionals are aware of their own practice in relation to recommended practice.
**20**	Team communication[4]	Type, quantity, communication flow between the pharmacy's staff around MAS.
**21**	Change objectives and their feedback[2]	The degree to which implementation objectives have been defined, communicated and achieved by the members of the pharmacy. (*Eg. Objectives for the provision of MAS, objectives set to solve detected barriers, target no. of patients)*
**22**	Monitoring and feedback[2]	The extent to which monitoring and feedback are needed at organisational level and available to sustain necessary changes.
**23**	Priority (relative) perception[1,2]	Perception shared by the pharmacy's workers about the importance of the implementation of MAS.
**24**	Culture[1]	Expectations and shared values of all the pharmacy's members.
**26**	Structural characteristics[1]	Pharmacy design, age, size and maturity in relation to the provision of MAS.
**27**	Resource availability[2]	The extent to which the resources that are needed to adhere are available.
**28**	Non-financial incentives[2]	The extent to which individuals have non-financial incentives to adhere (e.g. personal recognition, CPD).
**28.1**	Financial incentives (service profitability)[2]	The extent to which individuals have financial incentives or disincentives to adhere (e.g. ability to earn a profit from MAS).
**29**	External support[1,2]	Measure to which a pharmacy receives the external support required for practice change. *(Eg. Facilitator)*
**30**	Internal supporters and oponents[2]	Support provided by the pharmacy staff members for the implementation of MAS.<br>(Ex.: help from peers or co-workers, time needed to provide the service, etc.)
**31**	Readiness indicators[1]	Indicators inside of the pharmacy that show its commitment to the implementation of the service.
**32**	Leadership engagement[1]	Commitment, involvement, capability and responsibility of the head of the pharmacy towards implementing MAS.

**32.1**	Capacity to plan change[2]	The extent to which the targeted healthcare professionals have the capacity to plan necessary changes in order to adhere.
**32.2**	Business planning associated with change[4]	The extent to which leadership/team alter the strategic direction of the business to accommodate for introduced changes.
**33**	Customer needs[2]	Real or perceived needs and demands of the patients.
**35**	Relationship with pharmacists[1]	Relationship with other pharmacists to comment and share information in regards to MAS.
**36**	Relationship with physicians[1]	Working relationships established between the pharmacy and its pharmacists and physicians within its surroundings.
**37**	Physicians' awareness and observability[2]	Perception and knowledge of physicians on the necessity of providing MAS through pharmacists.
**38**	Patients' awareness and observability[2]	Patients' background knowledge on the necessity of providing MAS through pharmacists, and their own need of receiving it.
**39**	Competitor pressure[4]	The extent to which competition is affecting the pharmacy.
[1] Consolidated Framework of Implementation Research		
[2] TICD checklist		
[3] The Theoretical Domains Framework		
[4] New factors found in data		
