# Supplementary material for: Co-design and feasibility of a pharmacist-led minor ailment service
Source: BMC Health Serv Res. 2021 Jan 22;21:80. doi: 10.1186/s12913-021-06076-1 (PMC7821549; doi:10.1186/s12913-021-06076-1)
Supplement: Supplementary file 6 — Additional file 6. Example of completed facilitators database [file 12913_2021_6076_MOESM6_ESM.pdf]

Additional file 6 Example of completed facilitators database

Pharmacy no.	Facilitator	Visit no.	Barrier identified	Specific strategy chosen	Result
Pharmacy 1	1	V1	19. Workflow (Team processes)	2.4.7 Outline benefits (using opinion)	Resolved
Pharmacy 1	1	V1	19. Workflow (Team processes)	3.2.2 Suggest layout adaptation	Resolved
Pharmacy 1	1	V1	20. Team communication	3.4.3 Allocate primary champion	Resolved
Pharmacy 1	1	V1	20. Team communication	3.4.9 Allocate supporting champions	Resolved
Pharmacy 1	1	V1	18. Teamwork	3.4.1 Establish/ allocate roles	Resolved
Pharmacy 1	1	V1	20. Team communication	3.2.11 Utilise a meeting/ communication agenda	Unresolved
Pharmacy 1	1	V1	19. Workflow (Team processes)	3.5.2 Knowledge training	Resolved
Pharmacy 1	1	V1	30. Internal supporters and opponents	3.5.2 Knowledge training	Resolved
Pharmacy 1	1	V1	23. Priority (relative) perception	3.1.7 Use a prioritisation tool	Unresolved
Pharmacy 1	1	V1	33. Customer needs	3.1.8 Utilise goal-setting (SMART goals)	Resolved
Pharmacy 1	1	V1	19. Workflow (Team processes)	3.2.10 Adapt process/ procedures to new changes	Resolved
Pharmacy 1	1	V2	6. Resource use by staff	4.2.1 Provide constructive feedback	Unresolved
Pharmacy 1	1	V2	19. Workflow (Team processes)	1.2.1 Observations	Resolved
Pharmacy 1	1	V2	2. Time	1.2.1 Observations	Resolved
Pharmacy 1	1	V2	23. Priority (relative) perception	2.4.1 Address specific concerns	Unresolved
Pharmacy 1	1	V2	20. Team communication	3.2.9 Encourage regular communication among participants eg. daily huddles	Resolved
Pharmacy 1	1	V2	20. Team communication	3.4.6 Encourage collaboration and teamwork	Resolved
Pharmacy 1	1	V2	6. Resource use by staff	3.5.1 Skills/technical training	Resolved
Pharmacy 1	1	V2	23. Priority (relative) perception	2.4.6 Outline benefits (using evidence)	Unresolved
Pharmacy 1	1	V2	23. Priority (relative) perception	1.2.2 Interviews	Unresolved
Pharmacy 1	1	V2	23. Priority (relative) perception	2.2.1 Ask each person for their feedback regarding the change	Unresolved
Pharmacy 1	1	V2	23. Priority (relative) perception	2.4.3 Emphasize enhanced customer outcomes as opposed to poor practice as reason for change	Unresolved
Pharmacy 1	1	V2	6. Resource use by staff	2.5.3 Visual display (Poster)	Resolved

Pharmacy 1	1	V3	32. Leadership engagement	2.4.2 Compare audit results to network benchmarking.	Resolved
Pharmacy 1	1	V3	19. Workflow (Team processes)	3.2.2 Suggest layout adaptation	Resolved
Pharmacy 1	1	V3	23. Priority (relative) perception	1.2.7 Performance evaluation	Resolved
Pharmacy 1	1	V3	23. Priority (relative) perception	4.2.1 Provide constructive feedback	Resolved
Pharmacy 1	1	V3	23. Priority (relative) perception	4.2.2 Acknowledge success/ recognise /celebrate achievements	Resolved
Pharmacy 1	1	V3	23. Priority (relative) perception	2.5.4 Written document (email, letter etc.)	Resolved
Pharmacy 1	1	V3	23. Priority (relative) perception	3.1.8 Utilise goal-setting (SMART goals)	Resolved
Pharmacy 1	1	V3	23. Priority (relative) perception	4.1.6 Monitor agreed upon plan/ objectives	Resolved
Pharmacy 1	1	V3	23. Priority (relative) perception	4.1.7 Display progress chart	Resolved
Pharmacy 1	1	V4	23. Priority (relative) perception	4.2.3 Provide ongoing encouragement	Resolved
Pharmacy 1	1	V4	23. Priority (relative) perception	4.3.1 Ensure ongoing communication- via Email	Resolved
Pharmacy 1	1	V4	23. Priority (relative) perception	4.3.3 Ensure ongoing communication- Face to face	Resolved
