# Supplementary material for: Co-design and feasibility of a pharmacist-led minor ailment service
Source: BMC Health Serv Res. 2021 Jan 22;21:80. doi: 10.1186/s12913-021-06076-1 (PMC7821549; doi:10.1186/s12913-021-06076-1)
Supplement: Supplementary file 7 — Additional file 7. PRISMA diagrams and the results of the quality assessment process [file 12913_2021_6076_MOESM7_ESM.pdf]

Additional file 7 PRISMA diagrams and the results of the quality assessment process

PRISMA table of systematic searches

	**Headache**	**Migraine**	**Dysmenorrhea**	**Low Back Pain**	**Reflux**	**Common cold**	**Cough**
Records screened, duplicates removed	300	300	300	300	300	300	300
Title and abstract records excluded	168	203	261	293	212	292	270
Full-text guidelines assessed for eligibility	132	97	39	7	89	8	30
Full-text guidelines excluded	117	87	38	6	77	6	28
Articles included in synthesis	15	10	1	1	12	2	2
Additional articles identified via references	0	0	1	1	1	0	0
Articles included in synthesis	15	10	2	2	13	2	2

Final list of guidelines included eg. Headache, migraine, dysmenorrhoea, low back pain

Types of pain	Identification number	Clinical guideline	Country	Publication year/amended year
Headache	1	Therapeutic Guidelines	Australia	2014
	2	Guideline for primary care management of headache in adults	Canada	2015
	3	Primary care management of headache in adults	Canada	2016
	4	University of Saskatchewan Minor Ailment Scheme	Canada	2017
	5	Nova scotia Emergency Service Care Headache	Canada	2015
	6	Centre for Effective Practice	Canada	2016
	7	The National Institute for Health and Care Excellence (NICE)	UK	2017
	8	Nottinghamshire Area Prescribing Committee	UK	2016
	9	Diagnosis, assessment and management of Headache	UK	2016
	10	South and West Devon Formulary and Referral	UK	2017
	11	North West Headache Management Guideline	UK	2015
	12	Rotherham Guideline	UK	2013
	13	Ibuprofen and Tension Type Headache	UK	2014
	14	Telford and Wrekin Clinical Group: Headache in Over 12's Prescribing Guideline	UK	2015
	15	Gloucestershire Community Pharmacy Minor Ailments	UK	2015
Migraine	1	Therapeutic Guidelines	Australia	2014
	2	Guideline for primary care management of headache in adults	Canada	2015
	3	Clinical Protocol for Migraine	Canada	2015
	4	Nottinghamshire Area Prescribing Committee Adult Headache Pathway	UK	2016
	5	Northern East Adult Headache Management	UK	2016

	6	Dudley Clinical commissioning group Clinical Guideline for management of headache	UK	2016
	7	Buckinghamshire Healthcare Headache Management Guidelines for Adults	UK	2016
	8	South Western Ambulance Service	UK	2014
	9	Bath Area Joint Formulary: Anti-migraine drugs	UK	2017
	10	Telford and Wrekin Clinical Group: Headache in Over 12's Prescribing Guideline	UK	2015
Dysmenorrhea	1	Therapeutic Guidelines	Australia	2014
	2	University of Saskatchewan Minor Ailment Scheme	Canada	2017
Low back pain	1	Therapeutic Guidelines	Australia	2017
	2	The National Institute for Health and Care Excellence (NICE)	UK	2016

Guideline components

eg. Reflux guidelines

Guideline identification	Common symptoms	Diagnosis/ assessment	Red flags	Pharmacological	Non-pharmacological
1	+	+	+	+	+
2	+	(+/-)	+	+	(+/-)
3	+	+	+	+	+
4	+	+	(+/-)	+	+
5	+	+	+	+	+
6	+	+	+	+	+
7	+	+	+	+	+
8	+	+	+	+	(+/-)
9	+	+	+	+	+

10	+	+	+	(+/-)	(+/-)
11	-	(+/-)	+	-	+
12	-	+	+	(+/-)	+
13	+	+	+	+	+

AGREE II Guideline appraisal

eg. Reflux guidelines

Guideline identification	Domain 1 (Scope and purpose)	Domain 2 (Stakeholder involvement)	Domain 3 (Rigour of development)	Domain 4 (Clarity of presentation)	Domain 5 (Applicability)	Domain 6 (Editorial independence)
1	11%	11%	17%	56%	8%	50%
2	17%	17%	4%	56%	0%	0%
3	100%	61%	25%	89%	17%	0%
4	56%	28%	15%	89%	8%	0%
5	39%	11%	23%	83%	8%	0%
6	89%	22%	23%	89%	33%	17%
7	100%	100%	100%	100%	100%	25%
8	28%	17%	13%	94%	0%	0%
9	17%	17%	6%	56%	0%	0%
10	72%	61%	23%	100%	13%	0%
11	44%	17%	10%	89%	0%	0%
12	44%	33%	6%	89%	0%	0%
13	72%	89%	67%	94%	4%	33%
