# Supplementary material for: Co-design and feasibility of a pharmacist-led minor ailment service
Source: BMC Health Serv Res. 2021 Jan 22;21:80. doi: 10.1186/s12913-021-06076-1 (PMC7821549; doi:10.1186/s12913-021-06076-1)
Supplement: Supplementary file 8 — Additional file 8. HealthPathways [file 12913_2021_6076_MOESM8_ESM.pdf]

HealthPathways

For Pharmacists-Reflux Pharmacy Protocol

This protocol is for community pharmacists participating in the trial of a collaborative minor ailments service. The protocol offers best practice advice on the care of adults aged ≥ 18 years with symptoms suggestive of reflux.

Disclaimer

About

About reflux

About Reflux

- Reflux of gastric contents into the oesophagus is a normal physiological process.
- Heartburn is a burning sensation rising from the epigastrium toward the neck. It is usually caused by reflux of acid into the oesophagus and may be associated with regurgitation.
- 15 to 20% of adults experiencing heartburn at least once a week.
- Persistent symptoms, occurring more than twice weekly, are considered to be gastroesophageal reflux disease (GORD).
- Causes may include:
  - relaxation of the lower oesophageal sphincter.
  - increased lower abdominal pressure
  - delayed gastric emptying
  - impaired oesophageal clearance

Red flags

Send to emergency department immediately if:

- Black, tarry stool
- Vomiting that is persistent, protracted, or contains blood.
- Crushing chest pain radiating to the back, neck, jaw or arms
- Chest discomfort exacerbated by exercise.
- Trouble breathing or feeling faint
- Severe or disabling pain

Consultation

1. Take a history:
   - symptoms

     #### Symptoms

     - Location and frequency of pain
     - Nature and severity of pain. If gnawing, sharp, or stabbing it is unlikely to be reflux.
     - Radiation: referred pain might indicate cardiovascular origin e.g., radiation to jaw, neck, or left arm.
     - Associated symptoms: black and tarry stools (indicate a bleed in the gastrointestinal tract).

   - classification/severity

     #### Classification or severity

     - Severe: patient waking at night and symptoms significantly impact quality of life.

- Moderate: symptoms are frequent, intense and prolonged, and impacting quality of life.
- Mild: symptoms are not overly bothersome, although can occur frequently or infrequently.

- risk factors

Risk factors

- Overweight or obese
- Pregnancy
- Smoking
- Other medical conditions e.g., Crohn's disease, hypothyroidism, hypocalcaemia

- aggravating factors

Aggravating factors

Ask about:

- foods that can reduce lower oesophageal sphincter pressure e.g., fatty foods, garlic, onions, spearmint, peppermint, and alcohol.
- food and drinks that can make symptoms worse e.g., acidic or spicy food, caffeine, fruit juice.
- eating habits that can make symptoms worse e.g., overeating and eating "on the move".

- medications

Medications

- The use of medications which lower oesophageal pressure include:
  - calcium channel blockers
  - nitrates
  - anticholinergics e.g., benzhexol, benztropine, oxybutynin, tolterodine, tricyclic antidepressants, antipsychotics, and sedating antihistamines
  - dopaminergic medicines
  - nicotine replacement therapy (NRT)
  - progesterone
  - benzodiazepines
  - phosphodiesterase-5 (PDE-5) inhibitors e.g., sildenafil, tadalafil, vardenafil
  - beta-agonists e.g., salbutamol, terbutaline, eformoterol
- The use of medications which may cause or exacerbate oesophagitis include:
  - non-steroidal anti-inflammatory drugs (NSAIDS), including low-dose aspirin
  - bisphosphonates
  - iron
  - potassium chloride
  - tetracyclines
- The use of medications which may delay gastric emptying include:
  - narcotic analgesics
  - glucagon-like peptide-1 (GLP-1) analogues (e.g. exenatide, liraglutide)
  - anticholinergics.

2. Consider differential diagnosis.

Differential diagnosis

- Gastric or duodenal ulceration:
  - Can lead to gastrointestinal bleeding, perforation, and gastric obstruction.
  - Nearly 75 percent of the people who have gastric or duodenal ulcers don't have symptoms. In fact, these ulcers rarely cause severe symptoms.
  - Possible severe symptoms or signs include: black and tarry stool, vomiting blood, stabbing or gnawing pain, persistent and severe.
- Gastric cancer: Upper abdominal discomfort with other possible associated symptoms including nausea and vomiting, weight loss, black and tarry stool or vomiting blood.
- Oesophageal cancer: may present with symptoms of pain or difficulty swallowing, sensation of food sticking in oesophagus, unintended weight loss.
- Gallstones: Pain in right upper abdomen especially after a big meal.
- Ischaemic heart disease (IHD)
  - Nature of pain - tightness, burning, crushing, squeezing, constricting, vice-like, aching.
  - Location of pain: chest, shoulder(s), neck, arm(s), jaw, back
  - Associated symptoms: nausea, dizziness, cold sweat, shortness of breath, discomfort or pain in the upper abdomen (dyspepsia) and feeling unwell generally

3. Identify any concerning features and refer appropriately to general practitioner unless otherwise indicated.

Concerning features

- Anaemia
- Painful or difficult swallowing
- Sensation that food is 'sticking' in the throat
- Abdominal mass
- Severe or debilitating pain
- Treatment failure (using non-pharmacological and non-prescription measures)
- Adverse drug reaction suspected or suspected medication induced reflux e.g., from bisphosphonate
- Unexplained cough, dyspnoea, or hoarseness
- Long-standing change in bowel habit
- Aged < 18 years, or > 55 years with new onset or undiagnosed symptoms
- Unexplained weight loss associated with gastrointestinal symptoms
- Symptoms that are severe or frequent (such as awaking patient during night), or if symptoms have a significant impact on daily life

Treatment

1. For all patients provide non-pharmacological support.

Non-pharmacological support

- If symptoms are considered to be a minor ailment condition, proceed with a standardised management approach.
- Provide each patient a PSA Self-Care Fact Card.
- For all patients regardless of the severity or frequency:
  - Review and avoid trigger factors:
    - If patient is overweight or obese, advise weight loss.
    - Advise smoking cessation and alcohol intake reduction.
    - Refer to general practitioner to consider change or stop medicines known to cause or worsen reflux (as listed in the consultation).

- Provide nutrition advice:
  - Eat smaller, frequent meals.
  - Drink fluids between meals rather than with meals.
  - Not eating large meals near bedtime.
  - Avoid trigger foods/drinks.
  - Reducing alcohol intake.
- Simple measures:
  - Avoid lying down for up to 3 hours after eating.
  - Raising the bed head (if symptoms occur at night).

2. Consider pharmacological options and be aware of specific considerations.

Specific considerations

In elderly: avoid constipating products.

During pregnancy:

- Trial non-pharmacological recommendations initially.
- If drug therapy is required:
  - Antacids are pregnancy category A, and are the treatment of choice for pregnant women with GORD.
  - H2-antagonists, ranitidine is Category B1.
  - PPIs should only be used if antacids and H2-antagonists have not been effective.
  - Esomeprazole, lansoprazole, omeprazole, and pantoprazole are category B3, and rabeprazole is category B1.
  - If a PPI is required during pregnancy, omeprazole has the most data in humans and appears safe to use.

Manage medications according to symptom severity.

- mild infrequent (≤ 2 times a week)

Mild infrequent

- Antacids when required and/or H2-receptor antagonists (Ranitidine) for 14 days.
  - Combination antacids: dose according to label, up to 1 g elemental calcium daily. Liquid products are more effective, but less convenient, than solid products
  - Advise patient to:
    - Take between meals or at bedtime when symptoms occur or patient expects they might occur. Optimum antacid effect is achieved if taken 1 – 3 hours after meals.
    - Chew or suck before swallowing for the best effect.
    - Take antacids at least 2 hours away from other medicines by at least 2 hours.
  - Simple:
    - Sodium bicarbonate, potassium bicarbonate, calcium carbonate e.g., Quik-eze, Tums, Eno, Alka-seltzer.
    - Simple combinations: Rennie (calcium carbonate with magnesium carbonate).
    - Generally well tolerated but may experience some stomach distension, flatulence, belching.
  - Aluminium or Magnesium:
    - Aluminium hydroxide, magnesium hydroxide, magnesium trisilicate, magnesium sulfate, magnesium carbonate; e.g. Alu-tabs, Mylanta, Gaviscon Relief, Gastrogel.
    - Aluminium based side effect: Constipation.
    - Magnesium based side-effect: Diarrhoea.
  - Alginates: Combination with antacids e.g., Gaviscon, Mylanta heartburn relief.
- H2-receptor antagonist: If antacids do not give adequate relief, use Ranitidine:
  - 150 mg then repeat after 1 hour (Max daily dose 300 mg), or

        - 300 mg once daily.
        - Onset after 20-30min and last 4 to 12 hours.
    - If symptoms:
        - resolve, continue lifestyle recommendations and medication as required.
        - do not resolve, treat as per mild frequent or moderate reflux.

- mild and frequent (>2 times a week) or moderate

Mild and frequent or moderate

Trial a proton pump inhibitor (PPI) for 14 days.

- Dose: once daily 30 to 60 minutes before a meal.
- Advise patient:
    - Swallow whole with glass of water - do not crush or chew.
    - If their symptoms occur:
        - mainly during the day, to take the proton pump inhibitor before breakfast.
        - mainly at night, to take the proton pump inhibitor before the evening meal.
- It takes 2 to 3 days for the PPI to reach maximum effectiveness, and the patient may need adjuvant therapy with an antacid until then.
- Supply either:
    - Esomeprazole 20 mg daily.
    - Rabeprazole 10 mg daily.
    - Pantoprazole 20 mg daily.
    - Omeprazole 20 mg daily.

Referral

Arrange referral if symptoms are not considered to be a minor ailment condition or patient is presenting with red flags or concerning features. Determine patient's regular general practitioner or practice and arrange appropriate referral:

- Immediate emergency department referral:
    - Black, tarry stool
    - Blood in vomit
    - Vomiting that is persistent, protracted, or contains blood
    - Crushing chest pain radiating to the back, neck, jaw or arms
    - Chest discomfort exacerbated by exercise.
    - Trouble breathing or feeling faint
    - Severe or disabling pain
- Immediate general practitioner referral:
    - Known anaemia
    - Painful or difficult swallowing
    - Sensation that food is 'sticking' in the throat
    - Unexplained weight loss associated with gastrointestinal symptoms
    - Symptoms that are severe or frequent (such as awaking patient during night)
    - Significant impact on daily life
- 2 to 3 week general practitioner referral:
    - Unexplained cough, dyspnoea, or hoarseness
    - Longstanding change in bowel habit
    - Aged < 18 years, or > 55 years with new onset or undiagnosed symptoms
    - Adverse drug reaction suspected or suspected medication induced reflux e.g., from bisphosphonate
    - Symptoms that show no improvement after 2 weeks of proton pump inhibitor (PPI) therapy
    - Symptoms persist or relapse frequently

Documentation and general practitioner feedback

- Ensure that the patient:

- has read and signed the Patient Information and Informed Consent Form (to be kept at the pharmacy)
- has completed the EURO-QOL EQ-5D questionnaire (hard copy to be kept at the pharmacy)
- is informed of follow-up phone call conducted by the research team

- Provide date and time to patient of phone call (14 days after consultation in the pharmacy)
- Document the consultation by recording relevant information of the consultation on the data collection spreadsheet (for research purposes)
- Provide an electronic summary to the patient's regular general practitioner.

Provide an electronic summary to the patient's regular general practitioner.

- For all patients, provide a summary of the consultation, including non-prescription medicines provided.
- If referral was provided, indicate the criteria for referral.

Visit Health Link connect portal

Information

Patient information

- Gastroenterological Society of Australia – Heartburn (Oesophageal Reflux) Information Sheet
- Nice – GORD and Dyspepsia in Adults: Investigation and Management
- Healthdirect – GORD-Reflux
- Better Health Channel – Indigestion
- Choosing Wisely Australia – Heartburn and Reflux: Manage Your Medicine
- NPS – Your Heartburn and Reflux Management Plan
- Mothersafe – Heartburn in Pregnancy and Breastfeeding

Clinical resources

- Disorders of the oesophagus [revised 2017 Mar]. In: eTG complete [Internet]. Melbourne: Therapeutic Guidelines Limited; 2016 Mar.
- Australian Medicines Handbook 2017 (online). Adelaide: Australian Medicines Handbook Pty Ltd; 2017 Jan.
- National Institute for Health and Clinical Excellence (2014) - Gastro-oesophageal reflux disease and dyspepsia in adults: investigation and management. NICE guideline
- Rutter P, Newby D. Community pharmacy: symptoms, diagnosis and treatment. 2016; 3e
- Camilleri M, Parkman HP, Shafi MA, et al. Clinical guideline: management of gastroparesis. Am J Gastroenterol 2013;108(1):18–38
- Pharmaceutical Society of Australia. Guidance for provision of a pharmacist only medicine – proton pump inhibitors (PPIs). In: Sansom LN, ed. Australian pharmaceutical formulary and handbook. 23rd edn. Canberra: PSA; 2015.

Page Information

Information about this HealthPathways document (412706):
Document Owner: Not assigned (see Who's Who)
Last Updated:
Next Review:
Keywords:

Have you read the disclaimer?
© 2008-2018 WentWest Ltd

No content in this website may be reproduced or transmitted in any form or by any means, electronic, mechanical, recording, or otherwise, without the prior written approval of WentWest Ltd, and Canterbury District Health Board (where the original source of the content was from Canterbury District Health Board). For information on obtaining permission for reproduction or transmission of the content please contact info@healthpathways.org.nz.

HealthPathways

For Pharmacists - Common Cold Pharmacy Protocol

This protocol is for community pharmacists participating in the trial of a collaborative minor ailments service. The protocol offers best practice advice on the care of adults aged ≥ 18 years with symptoms suggestive of common cold.

Disclaimer

Red Flags

Send to emergency department immediately if:

- chest pain greater than mild retrosternal pain.
- severely unwell e.g., marked lethargy, shortness of breath.

About

About common cold

About common cold

- People on average suffer between 3 and 12 colds per year, depending on their age.
- In Australia, the peak times for the common cold are spring and winter.
- Most cold symptoms are caused by a virus.
- Most colds resolve within a week, but it is not unusual for a common cold to last for 14 days or more.
- The nature and severity of symptoms are influenced by factors including causative agent, patient's age, and underlying medical conditions.

Consultation

1. Consider typical symptoms and presentation.

Typical symptoms and presentation

- Sore throat and sneezing, followed by profuse nasal discharge and congestion.
- Cough commonly follows.
- Headache, mild to moderate fever (< 38.5 degrees Celsius), and general malaise might be present.
- Duration 7-10 days, peak around 72 hours.

2. Identify any:
   - concerning features and refer appropriately to general practitioner unless otherwise indicated.

Concerning features

- Symptoms suggestive of sinusitis:
  - Sinus tenderness
  - Symptoms last > 7 days
  - Thick mucopurulent discharge
  - Persistent headache
  - Radiation of pain to teeth and ears
- Symptoms suggestive of otitis media:
  - Significant ear pain
  - Reduced hearing
  - Fever
  - Headache
- Symptoms suggestive of influenza:

- Rapid onset over a few hours
- Fever
- Myalgia
- Loss of appetite
- Lethargy
- Headache

- High fever at onset of illness, or fever lasting longer than 3 days
- Worsening symptoms after initial improvement
- Vulnerable patient groups such as the elderly or immunocompromised
- Duration of illness > 7 - 10 days
- Excessive use of intranasal decongestants (rebound congestion)
- Toothache > 1 week, refer to dentist

- atypical symptoms suggestive of allergic rhinitis.

Atypical symptoms

- Perennial
- Sneezing
- Itching
- Lack of fever and aches and pains.

3. Ask about:
   - general medical history.
   - medications.

Treatment

1. Uncomplicated acute viral rhinosinusitis usually resolves in 7 to 10 days without treatment. For all patients provide non-pharmacological support.

Non-pharmacological support

If symptoms are considered to be a minor ailment condition, proceed with a standardised management approach.

Provide each patient a PSA Self-Care Fact Card.

- Advise the following:
  - rest
  - fluids
  - good hygiene to prevent spreading:
    - use disposable tissues rather than handkerchiefs
    - wash hands frequently (washing for 10 seconds, rinsing for 10 seconds, and then drying with a paper towel)
    - avoid sharing hand towels, cups, glasses and cutlery
    - avoid touching nose
    - cover mouth when sneezing or coughing
- Consider saline nasal sprays or irrigations which may have benefits for relieving symptoms of acute URTIs. Safe in pregnancy.[1]
- Steam inhalations:[2]
  - have not shown any consistent benefits in the treatment of the common cold and are not recommended in routine treatment of these symptoms.
  - no added benefit from additives e.g.,menthol, eucalyptus, pine
  - no evidence of worsening

  Caution: scalding, irritation of lips, nose, and eyes

2. Consider pharmacological options for specific symptoms with an awareness of special considerations:

Specific considerations

- Single ingredient products are preferable
- Do not combine a cough suppressant with an expectorant
- Avoid fixed dose combinations containing paracetamol: doses may be insufficient for antipyretic or analgesic effects, leading to risk of overdosage with other paracetamol products.
- Avoid sympathomimetics in cough e.g., pseudoephedrine:
  - Therapeutic value is doubtful.
  - Adverse effects include CV and CNS stimulation.
- Check combination products for adequate doses, as ingredients may be subtherapeutic.
- For pregnancy:
  - Single agents are Category A.
  - Combination products with a sympathomimetic are category B2 and should be avoided.
  - It is safe to use paracetamol and saline nasal sprays.
- For diabetics: Use sugar free preparations and monitor blood glucose during infection.

- Aches, pain, and fever

Aches, pain, and fever

Provide simple analgesics:

- Paracetamol
  - 500 mg - 1 g (1-2 tablets) every 4 hours as required
  - Maximum of 4 g (8 tablets) in 24 hours
  - Caution with other paracetamol containing products: combination cold and flu, Lemsip, Panadol osteo
- Aspirin
  - 300 - 500 mg (1 tablet) to 900 -1000 mg (2 or 3 tablets) every 4 to 6 hours as required
  - Maximum of 4 g (or 4 doses) in 24 hours
- Ibuprofen
  - 400 mg (2 tablets), then 200 to 400 mg (1-2 tablets) every 4 hours as required
  - Maximum of 1200 mg (6 tablets) in 24 hours

- Nasal congestion

Nasal congestion

Topical decongestants (nasal drops or spray)

- Onset 5 - 10 minutes
- Duration 6 - 12 hours
- Adverse drug reactions: local irritation and rebound congestion
- Nasal decongestant sprays should only be used for 3 days. If symptoms of nasal congestion are due to excessive use of intranasal decongestants (rebound congestion), wean or cease medications and advise patient that it may take several weeks to reverse.
- Consider:
  - Oxymetazoline: Nasal spray 0.05%: 1 or 2 sprays into each nostril up to 3 times daily, or
  - Xylometazoline:
    - Nasal spray 0.1%: 1 spray into each nostril up to 3 times daily.

- Nasal drop 0.1%: 2 or 3 drops into each nostril up to 3 times daily, or
- Tramazoline: Nasal spray, 1 or 2 sprays into each nostril up to 4 times daily.

Oral decongestants

- Onset 30 - 60 minutes
- Duration 3 - 4 hours
- Adverse drug reactions: hypertension, altered blood glucose control, insomnia.
- Limit use to 3 - 5 days
- Fixed-dose combinations are not listed as they are not recommended.
- Consider:
  - Pseudoephedrine
    - Oral, 60 mg every 4 - 6 hours (maximum 240 mg daily).
    - Controlled release tablet, 120 mg every 12 hours until symptoms improve, then reduce to 120 mg once daily, if needed.
  - Phenylephrine: Oral, 10 mg every 4 hours

- Runny nose - Note that use of non-sedating antihistamines or intranasal corticosteroids is not indicated.

Runny nose

Topical anticholinergics (spray)

- Ipratropium (Atrovent Nasal, Atrovent Nasal Forte):
- Dose: Initially 88 mcg (2 - 4 sprays) into each nostril 3 or 4 times daily, reducing as rhinorrhoea improves. use for up to 4 days

Oral antihistamines (First generation / sedating)

- When used on their own, there is no evidence of any significant effect in recovery from the common cold, although there is a small benefit on symptoms of rhinorrhoea and sneezing and increased sedation. When used in combination with decongestants there is a small reduction in nasal symptoms.[3]
- Duration 4 - 6 hours
- Adverse drug reactions: anticholinergic (dry mouth, constipation, precaution in glaucoma and prostate enlargement)
- Dexchlorpheniramine (Polaramine): Adult, child > 12 years, oral 2 mg 4 times daily.
- Chlorpheniramine (in compound cold, flu and sinus preparations)

- Cough

Referral

Determine patient's regular general practitioner or practice and arrange appropriate referral:

- Immediate emergency referral:
  - Chest pain greater than mild retrosternal pain
  - Severely unwell e.g., marked lethargy, shortness of breath
- Immediate general practitioner referral:
  - Symptoms suggestive of sinusitis e.g., sinus tenderness, symptoms last > 7 days, thick mucopurulent discharge, persistent headache, and radiation of pain to teeth and ears.
  - Symptoms suggestive of otitis media e.g., significant ear pain, reduced hearing, fever, and headache.
  - Symptoms suggestive of influenza e.g., rapid onset over a few hours, fever, myalgia, loss of appetite, lethargy, and headache.
  - Worsening symptoms after initial improvement
  - Vulnerable patient groups such as the elderly or immunocompromised
  - No symptom improvement despite treatment and duration of illness > 7-10 days
  - Prolonged or high fever (39 degrees Celsius) at the onset of illness and lasting longer than 3 days

- 1 - 3 day general practitioner referral: Excessive use of intranasal decongestants (rebound congestion).
- If toothache, refer to dentist.

Documentation and general practitioner feedback

- Ensure that the patient:
  - has read and signed the Patient Information and Informed Consent Form (to be kept at the pharmacy)
  - has completed the EURO-QOL EQ-5D questionnaire (hard copy to be kept at the pharmacy)
  - is informed of follow-up phone call conducted by the research team
- Provide date and time to patient of phone call (14 days after consultation in the pharmacy)
- Document the consultation by recording relevant information of the consultation on the data collection spreadsheet (for research purposes)
- Provide an electronic summary to the patient's regular general practitioner.

Provide an electronic summary to the patient's regular general practitioner.

- For all patients, provide a summary of the consultation, including non-prescription medicines provided.
- If referral was provided, indicate the criteria for referral.

Visit Health Link connect portal

Information

For patients

NPS:

- Colds, Coughs, and Flu: What You Can Do
- Cough, Colds, and Sore Throats Fact Sheet

For health professionals

- Acute rhinosinusitis [revised 2017 Mar]. In: eTG complete [Internet]. Melbourne: Therapeutic Guidelines Limited; 2014 Nov.
- Australian Medicines Handbook 2017 (online). Adelaide: Australian Medicines Handbook Pty Ltd; 2017 Jan.
- Rutter P, Newby D. Community Pharmacy: Symptoms, Diagnosis and Treatment. 2016; 3e
- University of Sydney Faculty of Sydney – A Cost-Benefit Analysis of Pharmacist Only (S3) and Pharmacy Medicines (S2) and Risk-Based Evaluation of the Standards

Sources

References

1. King D, Mitchell B, Williams C, Spurling G. Nasal saline irrigation for acute upper airway infection symptoms. Cochrane Database of Systematic Reviews. 2015;(4).
2. Singh M, Singh M, Jaiswal N, Chauhan A. Cochrane. [place unknown]: Cochrane; Heated, humidified air for the common cold. 2017.
3. De Sutter A, Saraswat A, van Driel M. Antihistamines for the common cold. Cochrane Library. 2015.

Page Information

Information about this HealthPathways document (418387):
Document Owner: Not assigned (see Who's Who)
Last Updated:
Next Review:
Keywords:

Have you read the disclaimer?
© 2008-2018 WentWest Ltd

No content in this website may be reproduced or transmitted in any form or by any means, electronic, mechanical, recording, or otherwise, without the prior written approval of WentWest Ltd, and Canterbury District Health Board (where the original source of the content was from Canterbury District Health Board). For information on obtaining permission for reproduction or transmission of the content please contact info@healthpathways.org.nz.

HealthPathways

For Pharmacists - Cough Pharmacy Protocol

This protocol is for community pharmacists participating in the trial of a collaborative minor ailments service. The protocol offers best practice advice on the care of adults aged ≥ 18 years with symptoms suggestive of cough.

Disclaimer

About

About cough

About Cough

- Cough is the fourth most common reason for visiting a general practitioner.[1]
- Acute coughs are most often associated with viral infection and are one of the most common presentations in pharmacies.
- Common causes of an acute cough include infection, allergies, and upper airways cough syndrome (previously called post-nasal drip). Viral upper respiratory tract infections are the most common cause of an acute cough at all ages.
- Coughs can be either acute or chronic. Australian guidelines define cough as follows:[2]
  - Acute cough: cough duration < 2 weeks
  - Chronic persistent cough: cough duration > 8 weeks
- Viral coughs typically present with sudden onset and associated fever. Sputum production is minimal and symptoms are often worse in the evening. Associated cold symptoms are also often present and last between 7 and 10 days. Duration of longer than 14 days might indicate a 'post-viral cough' or a bacterial secondary infection, but this is clinically difficult to distinguish without analysing sputum samples.

Red Flags

Send to emergency department if chest pain greater than mild retrosternal pain.

Consultation

1. Take a history:
   - symptoms

Symptoms

Coughs can be described as either productive (chesty, wet, phlegmy) or non-productive (dry, tight, tickly). If patient says that they are not producing sputum but 'can feel it on their chest' it is probably productive.

- Sputum colour:
  - Mucoid (clear and white) is normally of little consequence and suggests that no infection is present.
  - Yellow, green or brown sputum normally indicates infection. However, mucopurulent sputum is probably caused by a viral infection and does not require automatic referral.
  - Haemoptysis can be rust coloured (pneumonia), pink tinged (left ventricular failure), or dark red (carcinoma).
- Nature of sputum
  - Thin and frothy suggests left ventricular failure
  - Thick, mucoid to yellow can suggest asthma
  - Offensive, foul-smelling sputum suggests either bronchiectasis or lung abcess
- Onset of cough: A cough that is worse in the morning suggests upper airways cough syndrome, bronchiectasis or chronic bronchitis

- Duration of cough:
  - Upper respiratory tract infection (URTI) cough can last for more than 3 weeks and is called a 'post-viral cough'. However, view coughs lasting longer than 2 weeks with caution because they might indicate more serious pathology.
  - The longer the cough is present, the more likely serious underlying pathology is responsible. For example:
    - 3 days duration: most likely an URTI
    - 3 weeks duration: most like acute or chronic bronchitis
    - 3 months duration: chronic bronchitis, tuberculosis and carcinoma become more likely

Periodicity: Adult patients with recurrent cough might have chronic bronchitis, especially if they smoke.

- patient age

Patient age

With increasing age conditions such as bronchitis, pneumonia, and carcinoma become more prevalent.

- smoking history

Smoking history

Patients who smoke are more prone to chronic and recurrent cough. Over time this might develop into chronic bronchitis and emphysema.

- relevant past medical history and social history

Relevant past medical history and social history

Ask about:

- Asthma
- COPD / bronchiectasis
- History of PE / DVT
- Medications e.g., ACEI
- Allergies

2. Identify any concerning features and refer immediately to general practitioner, as below.

Concerning features

- High fevers present with cough
- Haemoptysis
- Cough > 2 weeks or recurrent cough (especially smoker with > 20 year pack history)
- Pain on inspiration
- Shortness of breath, especially at rest or at night
- Sputum that is thick, yellow, green, grey, blood stained, rust coloured, or frothy pink
- Systemic symptoms e.g., fever, weight loss, lethargy
- Severe cough or spasms of coughing
- Smoker aged > 45 years with a new cough, altered cough, or cough with voice disturbance

3. Consider other possible diagnoses.

Other possible diagnoses

- Asthma: night-time cough and wheeze.
- Bronchitis: smoking adults. Cough becomes productive.
- Bronchogenic carcinoma: small amount of sputum, haemoptysis, continues despite smoking cessation, weight loss, malaise.
- Drug-induced: angiotensin converting enzyme inhibitors (ACEI) side effect.
- Gastro-oesophageal reflux disease: Cough worse when lying down.
- Heart failure: Elderly. Frothy or pink tinged sputum, shortness of breath, worse when lying down, fatigue, and oedema.
- Pneumonia: haemoptysis, high fever, pleuritic pain.
- Pulmonary embolism: pain on inspiration and shortness of breath.
- Tuberculosis: slow onset, haemoptysis.
- Upper airways cough syndrome (post nasal drip). Associated with: URTI (viral), allergy (sneezing, conjunctivitis, itching nasal cavity), and excessive throat clearing. Minimal sputum, worse at night.

Treatment

1. For all patients provide non-pharmacological support.

Non-pharmacological measures

- If symptoms are considered to be a minor ailment condition, proceed with a standardised management approach.
- Provide each patient a PSA Self-Care Fact Card
- Advise the following:
  - Rest
  - Fluids
  - Smoking cessation
  - Vocal hygiene measures e.g., avoid overuse of the voice and clearing the throat, and minimise coughing (taking sips of water with a hard swallow)
- Treat or remove remove underlying causes:
  - Upper airway cough syndrome (UACS) due to common cold symptoms or allergy
  - Cigarette smoke, cold air, other inhaled irritants
- For a dry cough trial simple linctus or hard lolly. These demulcents suppress coughing by forming a protective layer over sensory receptors in the pharynx.

2. Consider pharmacological options for specific symptoms with an awareness of special considerations:

Specific considerations

- Single ingredient products are preferable
- Do not combine a cough suppressant with an expectorant
- Avoid fixed dose combinations containing paracetamol: doses may be insufficient for antipyretic or analgesic effects, leading to risk of overdosage with other paracetamol products.
- Avoid sympathomimetics in cough e.g., pseudoephedrine:
  - Therapeutic value is doubtful.
  - Adverse effects include CV and CNS stimulation.
- Check combination products for adequate doses, as ingredients may be subtherapeutic.
- For pregnancy:
  - Single agents are Category A.
  - Combination products with a sympathomimetic are category B2 and should be avoided.
  - It is safe to use paracetamol and saline nasal sprays.

- For diabetics: Use sugar free preparations and monitor blood glucose during infection.

- Productive cough
  - Mucolytics

Mucolytics

Promote expectoration of bronchial secretions by reducing viscosity.

Bromhexine[3]

- Adult dose: 8 -16 mg three times daily
- Pregnancy Category A
- Not recommended for patients with chronic bronchitis or COPD

  - Expectorants

Expectorants

Promote expectoration by reducing thickness and increasing sputum volume.

- Guaifenesin[4]
  - Adult dose: 200 - 400 mg four times a day
  - Pregnancy Category A
- Senega, ammonium salts, Ipecacuanha, Squill: no published evidence

- Non-productive cough – choose one of the following treatment approaches:
  - Treatment of non-productive cough with cough suppressants.

Treatment of non-productive cough with cough suppressants[5]

- Centrally acting to suppress cough reflex.
- Avoid use in productive cough, asthma and COPD.
- Consider:
  - Pholcodine
    - Non-opiate, less dependence
    - Adult dose: 10 - 15 mg 3 - 4 times daily (maximum dose 60 mg per day)
    - Adverse drug reactions: possible sedation
    - Pregnancy Category A
  - Dextromethorphan
    - Non-opiate, less dependence
    - Adult dose: 10 mL up to four times a day
    - Drug interaction with serotonergic agents e.g., antidepressants (MAOIs, SSRIs, St John's Wort)
    - Pregnancy: Safe to use
  - Dihydrocodeine
    - Synthetic derivative of codeine
    - Potential for abuse
    - Adult dose: 5 - 10 mL every 4 to 6 hours as required.
    - Adverse drug reactions: sedation
    - Pregnancy: safe to use

  - First generation antihistamines

First generation antihistamines

Anticholinergic and/or sedative effects may decrease cough by drying up secretions.

- May be useful if cough is associated with post nasal drip
- Primarily used for night-time cough or other symptoms e.g., allergic rhinitis
- Avoid if cough is productive
- In non-allergic cough there is no evidence of efficacy for second and third generation (less sedating) antihistamines e.g., fexofenadine, loratadine, desloratadine, cetirizine, levocetirizine.
- Consider:
  - Diphenhydramine (in combinations)
    - Schedule 3: Benadryl Original (with ammonium chloride)
    - Adult dose: 25 mg (10 mL) every 4 hours as required. (Maximum 6 doses in 24 hours).
    - Precautions: narrow angle glaucoma, prostate enlargement, asthmatics, elderly aged > 60 years
    - Pregnancy Category A
  - Dexchlorpheniramine
    - Schedule 3: Polaramine
    - Adult dose: 2 mg every 6 hrs
    - Precautions: narrow angle glaucoma, prostate enlargement, asthmatics, elderly > 60 years
    - Pregnancy Category A (do not use in third trimester)
  - Brompheniramine (in combinations)
    - Schedule 2: Demazin Cough & Cold Relief (with phenylephrine and dextromethorphan)
    - Adult dose: 10 mL every 4 hours as required. (Maximum 6 doses in 24 hours).
    - Pregnancy Category B2

Referral

Determine patient's regular general practitioner or practice and arrange appropriate referral:

- Immediate emergency referral: chest pain greater than mild retrosternal pain.
- Immediate general practitioner referral:
  - High fevers present with cough
  - Haemoptysis
  - Cough > 2 weeks or recurrent cough (especially smoker with > 20 year pack history)
  - Pain on inspiration
  - Shortness of breath, especially at rest or at night
  - Sputum that is thick, yellow, green, grey, blood stained, rust coloured, or frothy pink
  - Systemic symptoms e.g., fever, weight loss, lethargy
  - Severe cough or spasms of coughing
  - Smoker aged > 45 years with a new cough, altered cough, or cough with voice disturbance
- 2 - 3 week general practitioner referral:
  - Smoker with > 20 pack year smoking history.
  - Medication induced (Angiotensin converting enzyme inhibitors (ACEI) causing cough)

Documentation and general practitioner Feedback

- Ensure that the patient:
  - has read and signed the Patient Information and Informed Consent Form (to be kept at the pharmacy)
  - has completed the EURO-QOL EQ-5D questionnaire (hard copy to be kept at the pharmacy)
  - is informed of follow-up phone call conducted by the research team

- Provide date and time to patient of phone call (14 days after consultation in the pharmacy)
- Document the consultation by recording relevant information of the consultation on the data collection spreadsheet (for research purposes)
- Provide an electronic summary to the patient's regular general practitioner.

Provide an electronic summary to the patient's regular general practitioner.

- For all patients, provide a summary of the consultation, including non-prescription medicines provided.
- If referral was provided, indicate the criteria for referral.

Visit Health Link connect portal

Information

For patients

NPS:

- Colds, Coughs, and Flu: What You Can Do
- Cough, Colds, and Sore Throats Fact Sheet

For health professionals

- Cough [revised 2017 Mar]. In: eTG complete [Internet]. Melbourne: Therapeutic Guidelines Limited; 2015 Mar.
- Australian Medicines Handbook 2017 (online). Adelaide: Australian Medicines Handbook Pty Ltd; 2017 Jan.
- The University of Sydney Faculty of Pharmacy – A Cost-Benefit Analysis of Pharmacist Only (S3) and Pharmacy Medicines (S2) and Risk-Based Evaluation of the Standards.
- Rutter P, Newby D. Community Pharmacy: Symptoms, Diagnosis and Treatment. 2016; 3e

Sources

References

1. Britt H, Miller GC, Charles J, Henderson J, Bayram C, Valenti L, et al. General practice activity in Australia 1999 – 00 to 2008 – 09 : 10 year data tables Bettering the Evaluation And Care of Health. General Practice. 2009;(26):167.
2. Gibson PG, Chang AB, Glasgow NJ, Holmes PW, Katelaris P, Kemp AS, et al. CICADA: Cough in children and adults: Diagnosis and assessment. Australian cough guidelines summary statement. Medical Journal of Australia. 2010;192(5).
3. Nesswetha W. Criteria of drug testing in industrial practice, demonstrated by a cough remedy. Arzneimittelforschung. 1967;17(10):1324-6.
4. Robinson R. Effectiveness of guaifenesin as an expectorant: a cooperative double-blind study. Curr Ther Res. 1977;22:284-296.
5. Smith S. Over-the-counter medications for acute cough in children and adults in ambulatory settings. Cochrane Database of Systematic Reviews. 2008;(1).

Page Information

Information about this HealthPathways document (418760):
Document Owner: Not assigned (see Who's Who)
Last Updated:
Next Review:
Keywords:

Have you read the disclaimer?

© 2008-2018 WentWest Ltd

No content in this website may be reproduced or transmitted in any form or by any means, electronic, mechanical, recording, or otherwise, without the prior written approval of WentWest Ltd, and Canterbury District Health Board (where the original source of the content was from Canterbury District Health Board). For information on obtaining permission for reproduction or transmission of the content please contact info@healthpathways.org.nz.

HealthPathways

For Pharmacists - Headache Pharmacy Protocol

This protocol is for community pharmacists participating in the trial of a collaborative minor ailments service. The protocol offers best practice advice on the care of adults aged ≥ 18 years with symptoms suggestive of two types of headache – migraine and tension headache.

Disclaimer

About

About headache

About headache

- Most headaches in adults are benign primary headaches, either tension-type headaches, or migraines.
- The most common types of benign primary headaches are:
  - tension headache.
  - migraine.
  - medication overuse headache.
  - cluster headache.
- Underlying serious pathology as a cause of headaches is rare, but may include:
  - Giant cell arteritis (GCA) – consider if new-onset headache in patients aged > 50 years.
  - Subarachnoid haemorrhage – sudden onset "thunderclap headache", like a "blow to the head", peak pain intensity within minutes, often occipital.
  - Meningitis or encephalitis – febrile, drowsy, neck stiffness, rash, recent infections.
  - Mass lesion e.g., tumour or abscess – headache when coughing, stooping, or straining with evolving increase in severity.
  - Stroke – rapid onset of neurological deficit with speech, limb or facial weakness.
  - Primary angle-closure glaucoma – monocular pain, red eye, visual disturbance, nausea.
  - Subdural haemorrhage – headache after trauma in elderly.

Emergency management

Send to emergency department immediately if:

- New headache with altered conscious state or visual deficit
- Monocular pain, red eye, visual disturbance
- Thunderclap headache – sudden onset
- Sudden loss of vision
- Altered conscious state
- Rapidly deteriorating cognitive state
- Acute onset of focal symptoms or signs e.g., nerve palsy, hemiparesis
- Fever and neck stiffness

Consultation

1. Take a history of:
   - pain

Pain

- Using a numeric pain scale, assess the level of pain the person is experiencing: 0 represents no pain, 10 the worst possible pain.

- A guide for severity and type of headache:
  - Tension headache: 2-5
  - Migraine: 4-7
  - Cluster: > 7
- Location and any radiation.
- Age of onset and duration / time of year.
- Exacerbating and relieving factors.

- associated symptoms e.g., nausea or vomiting, intolerance to bright lights, tears, or nasal congestion.
- relevant medical history e.g., previous malignancy, recent head injury, uncontrolled hypertension.
- possible triggers
- relevant family history e.g., of migraines
- social history and medications
- reassuring features

Reassuring features

- Recurrent episodic headaches with resolution of symptoms.
- Long history of headache without any neurological impairment.

2. Identify concerning features, and refer appropriately to general practitioner, as below.

Concerning features

- Recent onset, over weeks, "first, worst, or different"
- Increasing severity without remission
- Severe enough to wake the patient during the night or is present on waking
- Made worse by coughing, sneezing, bending, or exertion
- Previous malignancy especially lung, breast, or melanoma
- Head injury within the last month
- Aged > 60 years, especially if no headaches in the past
- Change in personality
- Vomiting without other obvious cause
- Compromised immunity
- Worsening headache with fever
- Uncontrolled hypertension

3. Assess for:
   - features of primary headaches in adults:
     - Tension-type

Tension type

- The most common chronic recurring headache.
- Duration: few hours up to 1 week.
- Severity: mild to moderate (pain scale: 2-5).
- Presents as either:
  - episodic (< 15 days per month), or
  - chronic (≥ 15 days per month).
- Typically bilateral, frontal or frontal-occipital.
- May radiate to the neck, with subjective neck stiffness.
- Sensation of pressure inside the head, or of a band around the head.
- May worsen as the day goes on.
- May be exacerbated by stress or "busyness" (often denied).
- Triggers can include excessive muscle contraction such as frowning or jaw-clenching from:

- poor posture at work, home and when driving
- bright lights, prolonged reading, loud noise
- stress, anxiety
- fatigue, emotional upsets, depression.

- Patient remains active.
- Episodic pattern may progress to "all day, every day" pattern.
- Potential to progress to medication overuse headache.

- Migraine

Migraine

Features:

- Duration – several hours or up to 3 days, recurrent episodes.
- Severity – moderate to severe (pain scale: 4-7).
- Onset is commonly in the teens, but can be any age.
- May wake the patient at night.
- Bending and coughing may exacerbate pain.

Ask about:

- severe unilateral or bilateral headache, often centred around the eye.
- nausea and vomiting.
- intolerance to bright lights or loud noises.
- exacerbation by movement and exercise.
- patient preference to lie in the dark.
- family history.
- If aura, ask about:
  - aura – usually precedes headache, but may be concurrent or, at times, occur with no headache. Aura usually lasts minutes or tens of minutes.
  - sensory aura – while visual aura is most common, sensory aura is also common e.g., unilateral paraesthesiae (arm or face).
  - other neurological features e.g., mild confusion or difficulty finding words, which are common.
  - unilateral weakness.

Known triggers:

- Dietary triggers e.g., chocolate, cheese, alcohol, caffeine.
- Change in habit e.g., missed meal, late night, travel.
- Strenuous physical activity, including sexual.
- Changes in the weather.
- Bright or flickering light
- Strong odours.
- Noise.
- Stress, or relaxation after stress.
- Interrupted sleep pattern.
- Hormonal changes e.g., combined oral contraceptive pill (COCP), pre-menstrual, or perimenopause.

Note: Most episodes do not have an obvious trigger.

- Medication overuse

Medication overuse

- Common and under-diagnosed.

- Can be caused by all medications used for immediate headache relief e.g., paracetamol, NSAIDs, caffeine, and codeine.
- Triptans are becoming a significant cause.
- Consider as a cause in all patients with chronic headaches.
- Develops in patients with a pre-existing primary headache disorder, usually migraine or tension-type headache.
- Features
  - Symptoms include either worsening of headache associated with frequent medication use, or worsening of headache with medication reduction.
  - Headache present ≥ 15 days per month.
  - Regular headache medication use for ≥ 3 months, at least 10 to 15 days per month.
  - Other features may include:
    - worse on waking.
    - aggravated by physical exercise.
    - nausea and other gastrointestinal symptoms.
    - restlessness, anxiety, irritability, and poor concentration.

- Cluster

Cluster

- Duration: 15 minutes to 3 hours if untreated. Occurs 1 to 8 times daily.
- Severity: Severe or very severe.
- Features
  - Mostly affects men aged 20 to 40 years (six males to every female).
  - May be missed, as uncommon in general practice.
  - Typically occurs in bouts of 6 to 12 weeks, every one to two years.
  - Often occurs at the same time of day and year, separated by periods of freedom from headache.
  - Most patients will develop longer remission intervals with increasing age.
- Pain characteristics (seen in at least 5 attacks)
  - Severe or very severe unilateral orbital, supraorbital or temporal pain.
  - Accompanying features:
    - Ipsilateral conjunctival injection and/or lacrimation
    - Nasal congestion and/or runny nose
    - Localised forehead and facial sweating
    - Constricted pupil and/or ptosis, eyelid oedema
    - Restlessness

- Other medication-related causes:
  - adverse effect: e.g., from dipyridamole, indomethacin, contraceptive pill, or nitrates.
  - withdrawal e.g., from analgesics or benzodiazepines.

Treatment

1. For tension headache or migraine, provide non-pharmacological support.

Non-pharmacological support for tension headache or migraine:

- If symptoms are considered to be a minor ailment condition, proceed with a standardised management approach.

- Recommend lifestyle adjustments as appropriate:
  - Reduce stress
  - Get adequate sleep and rest
  - Eat regularly
  - Encourage regular exercise and relaxation exercises e.g., yoga
- Recommend using a headache or migraine diary for 4 to 6 weeks to record frequency, intensity, suspected triggers, and the effectiveness of interventions.
- Review and avoid trigger factors.
- Tension headache: Provide each patient a PSA Self-Care Fact Card.
- Migraine:
  - Provide each patient a PSA Self-Care Fact Card.
  - Advise patient during an acute attack to:
    - rest in a dark, quiet room.
    - avoid movement and activity.

2. For medication-induced headaches, recommend monitored withdrawal of the offending medication.

Monitored withdrawal

- Full withdrawal of the offending medication is the only effective treatment.
- Referral as below.
- Advise patient that:
  - withdrawal symptoms may last approximately 2 -10 days but resolution of the problem may take much longer.
  - a 'prophylactic' drug may be required to cover withdrawal.
  - relapse is common.

3. Consider pharmacological options, with an awareness of specific considerations.

Specific considerations

Choice is based on side-effect profile, patients comorbidities, and previous tolerability to medication. All these medications have equal efficacy: diclofenac, ibuprofen, naproxen, paracetamol.

- Precautions:
  - Aspirin and NSAIDs – in patients who have asthma, increased bleeding risk, or heart failure.
  - Paracetamol in patients with hepatic failure
- Interactions: Aspirin and NSAIDs can interact with: sodium valproate, warfarin, lithium, diuretics, and antihypertensives.
- Supplying analgesics:
  - Avoid opioid analgesics.
  - Limit the use of analgesics to 2 to 3 times per week to reduce the risk of developing medication overuse headache.
  - Simple analgesics are usually preferred to using combination analgesics containing codeine (codeine can cause gastrointestinal symptoms and has a dependence risk):
    - No evidence of greater efficacy with the addition of codeine in doses less than 30 mg.
    - No evidence of increased analgesic effect with the addition of doxylamine.
  - Ensure that the doses given for headache are not sub-therapeutic.
  - No evidence of improved analgesia with the addition of codeine, caffeine, or sedating antihistamines e.g. doxylamine.
- Migraine:
  - Soluble forms may be preferable for migraine due to stasis of gastrointestinal tract.
  - Rapid absorption is required to be effective, therefore advise patient to take early in attack before nausea and reduced gastrointestinal

motility occur.
- Anti-emetics may improve absorption of analgesic drugs and may also reduce migraine pain by other mechanisms that are poorly understood.

- Sedating antihistamines may be of some benefit in patients who require sleep.

- Tension headache

Tension headache

Dispense simple analgesics as follows:

- Aspirin soluble 600 to 900 mg (2-3 tablets) orally. Repeat in 4 hours if required, or
- Ibuprofen 200 to 400 mg (1-2 tablets) orally. Repeat in 6 hours if required, or
- Diclofenac 12.5 to 25 mg (2 tablets) orally. Repeat in 6 hours if required, or
- Naproxen sodium 275 to 550 mg (1-2 tablets) orally. Repeat in 6 hours if required, or
- Paracetamol soluble 1 g (2 tablets) orally, 4 hourly. Maximum of 4g per day.

- Migraine

Migraine

Simple analgesics

Supply one of the following simple analgesics:

1. First line:
   - Aspirin soluble 600 to 900 mg (2-3 tablets) orally. Repeat in 4 hours if required, or
   - Ibuprofen 200 to 400mg (1-2 tablets) orally. Repeat in 6 hours if required, or
   - Diclofenac 50 to 100mg (2-4 tablets) orally. Repeat in 6 hours if required, or
   - Naproxen sodium 550 to 825mg (2-3 tablets) orally. Repeat in 6 hours if required. Maximum of 1375mg daily.
2. Second line: Paracetamol soluble (e.g. Panadol): 1 g (2 tablets) orally, 4 hourly. Maximum of 4g per day.

Anti-emetics

Consider for nausea and vomiting associated with migraine:

- Paracetamol 500 mg and metoclopramide 5 mg
  - Schedule 3: Metomax (caps), Anagraine (tabs), Trust Migraine Headache and Nausea Relief (tabs).
  - Adult dose: 1 - 2 tablets at the first sign of migraine, repeat every 4 hours if required. Maximum of 6 tablets or capsules per day
- Prochlorperazine 5 mg
  - Schedule 3: Nausetil, Nausrelief (tabs)
  - Adult dose: 1 -2 tablets two to three times a day.

Referral

- Determine patient's regular general practitioner or practice and arrange appropriate referral:
  - Immediate emergency referral:
    - New headache with altered conscious state or visual deficit
    - Monocular pain, red eye, visual disturbance
    - Thunderclap headache – sudden onset

  - Sudden loss of vision
  - Altered conscious state
  - Rapidly deteriorating cognitive state
  - Acute onset of focal symptoms or signs e.g. nerve palsy, hemiparesis
  - Fever and neck stiffness
- Immediate general practitioner referral:
  - Recent onset, over weeks, "first, worst, or different"
  - Increasing severity without remission
  - Severe enough to wake the patient during the night or is present on waking
  - Made worse by coughing, sneezing, bending, or exertion
  - Head injury within the last month
  - Aged > 60 years, especially if no headaches in the past
  - Change in personality
  - Vomiting without other obvious cause
  - Compromised immunity
  - Worsening headache with fever
  - Pain > 6 on scale 1-10
  - Uncontrolled hypertension
  - Suspected sinusitis or otitis media
- 2 to 3 week general practitioner referral:
  - Previous malignancy especially lung, breast, or melanoma
  - Headache unresponsive to adequate doses of analgesics
  - Headache lasting > 2 weeks
  - Symptoms indicating cluster headache
  - Frequency of > 6 headaches per month
  - Suspected medication overuse headache or withdrawal from certain medications
- If suspected dental abscess, recommend dental visit.
- Consider referring to other allied health professionals as appropriate e.g.,
  - for guidance with food triggers and nutrition, refer to dietitian
  - suspected visual involvement with no red flags, refer to optometrist
  - muscle or nerve tension, refer to physiotherapist or chiropractor.

Documentation and general practitioner feedback

- Ensure that the patient:
  - has read and signed the Patient Information and Informed Consent Form (to be kept at the pharmacy)
  - has completed the EURO-QOL EQ-5D questionnaire (hard copy to be kept at the pharmacy)
  - is informed of follow-up phone call conducted by the research team
- Provide date and time to patient of phone call (14 days after consultation in the pharmacy)
- Document the consultation by recording relevant information of the consultation on the data collection spreadsheet (for research purposes)
- Provide an electronic summary to the patient's regular general practitioner.

Provide an electronic summary to the patient's regular general practitioner.

- For all patients, provide a summary of the consultation, including non-prescription medicines provided.
- If referral was provided, indicate the criteria for referral.

Visit Health Link connect portal

Information

Patient information

- NPS - Headache diary

- Headache Australia
  - Support Group
  - What is headache?
  - Headache management

Clinical resources

- Headache [revised 2014 Feb]. In: eTG complete [Internet]. Melbourne: Therapeutic Guidelines Limited; 2011 Jul.
- Australian Medicines Handbook 2017 (online). Adelaide: Australian Medicines Handbook Pty Ltd; 2017 Jan.
- National Institute for Health and Care Excellence - Headaches in over 12s: diagnosis and management.
- Becker W. Guideline for primary care management of headache in adults .Canadian Family Physician August 2015, 61 (8) 670-679.
- Benrimoj SI. A Cost-Benefit Analysis of Pharmacist Only (S3) and Pharmacy Medicines (S2) and Risk-Based Evaluation of the Standards. 2005.
- Rutter P, Newby D. Community pharmacy: symptoms, diagnosis and treatment. 2016; 3e

Page Information

Information about this HealthPathways document (419548):

Document Owner: Not assigned (see Who's Who)

Last Updated:

Next Review:

Keywords:

Have you read the disclaimer?

© 2008-2018 WentWest Ltd

No content in this website may be reproduced or transmitted in any form or by any means, electronic, mechanical, recording, or otherwise, without the prior written approval of WentWest Ltd, and Canterbury District Health Board (where the original source of the content was from Canterbury District Health Board). For information on obtaining permission for reproduction or transmission of the content please contact info@healthpathways.org.nz.

HealthPathways

For Pharmacists-Lower Back Pain Pharmacy Protocol

This protocol is for community pharmacists participating in the trial of a collaborative minor ailments service. The protocol offers best practice advice on the care of adults aged ≥ 18 years with symptoms suggestive of low back pain.

Disclaimer

About

About lower back pain

Lower Back Pain

- About 80% of people will experience an episode of non-specific low back pain at some time in their lives.
- Most common between the ages of 35 and 55 years.
- Most patients with low back pain will have a non-specific local musculoskeletal condition with an acute and self-limited course.
  - Usually improves within 4 weeks.
  - No ongoing limitation in daily activities and can resume normal physical function.
- The prevalence of chronic severe, disabling pain increases with age.
- Acute pain if it persists < 12 weeks.
- Chronic pain if it persists > 12 weeks.
- Risk factors: heavy physical work, frequent bending, twisting, lifting, prolonged static postures.

Red Flags

Send to emergency department if any of the following are suspected:

- spinal cord compression or cauda equina syndrome
- spinal cancer
- spinal infection
- spinal fracture
- abdominal aortic aneurysm (AAA)
- severe neurological deficits with foot drop or unable to walk, or deficits at multiple levels.

Consultation

1. Assess for red flags:
   - spinal fracture: ask about recent significant trauma
   - spinal cord compression or cauda equina syndrome

     ### Cauda equina syndrome

     Ask about:

     - sudden onset of new urinary retention, and bowel disturbance (faecal incontinence or constipation) with saddle and perineal numbness.
     - lower limb symptoms which may be unilateral or bilateral and asymmetrical, including pain, weakness, and altered sensation.

   - spinal cancer

     ### Spinal cancer

     Ask about:

- history of cancer. Risk is increased these in patients.[1]
- symptoms including:
  - night pain
  - unexplained weight loss
  - aged > 50 years
  - persisting back pain > 12 weeks.

- spinal infection e.g., osteomyelitis of vertebra, discitis, extradural abscess, meningitis

Spinal infection

Ask about:

- new back pain with fever
- history of intravenous drug use
- immunosuppression
- recent infection
- recent epidural or spinal anaesthesia

- vertebral compression fracture

2. Ask about:
   - pain

Pain

- Location and any radiation.
- Onset: if insidious in onset, review with caution.
- Severity. Consider using:
  - Numerical rating scale from 0 (no pain) to 10 (worst pain).
  - Visual analogue scale e.g., 10cm unmarked line with "no pain" at the left end to "worst pain imaginable" at the right end.
  - Words to describe pain e.g., none, mild, moderate, severe, and worst possible pain.

- typical features of mechanical or simple lower back pain

Typical features of mechanical or simple lower back pain

- Usually localised to low back and buttocks.
- Typically intermittent, affected by posture and movement, improves with rest, and at night.
- Recovery is likely, and most people will improve in the first 12 weeks after the onset of symptoms.
- It is usually not possible to identify the actual cause of pain.

- relevant concerning medical and social history.

Relevant concerning medical and social history

- Recent bacterial infection e.g., UTI
- Immune suppression e.g., from corticosteroids, transplant, or HIV
- Osteoporosis
- Current or past cancer
- Intravenous drug use
- Major trauma e.g., vehicle accident or fall from height

3. Identify concerning features and refer appropriately to general practitioner, as below.

Concerning features

- Risk factors for vertebral compression fracture e.g., older age, history of osteoporosis, steroid use, menopause, or post chemotherapy.
- Risk factors for cancer e.g., history of cancer, unexplained weight loss, failure to improve after 1 month, age > 50 years.
- Young or older people e.g., aged < 20 years or > 55 years.
- Numbness.
- Lower back pain that is:
  - at multiple sites
  - worse when lying on back and / or at night time
  - of sudden onset
  - of high or severe level and causing disability at presentation
  - radiating away from lower back area
  - worse after sitting down for long periods – consider disc herniation
  - persistent and progressively worsening for more than 4 weeks
- Younger person with:
  - morning stiffness
  - improvement with exercise
  - alternating buttock pain
  - awakening due to back pain during the second part of the night

Treatment

1. Provide non-pharmacological support.

Non-pharmacological support

If symptoms are considered to be a minor ailment condition, proceed with a standardised management approach.

- In most patients with acute nonspecific low back pain, minimal intervention is required and symptoms resolve with appropriate patient education and reassurance of favourable prognosis.
- Provide each patient a PSA Self-Care Fact Card.
- Advise patient to:
  - stay active, starting with gentle movements e.g., water-based walking, land-based walking, gentle swimming, and floor stretches.
  - avoid prolonged bed rest.
  - maintain usual activities.
  - use gentle massage and application of heat and cold.
- Reassure patient that they are likely to make a full recovery.
- Promote prevention e.g, proper lifting techniques.
- Consider referral to an allied health professional e.g., physiotherapist or chiropractor.

2. Consider pharmacological options, with an awareness of specific considerations:

Specific considerations

- Opioids:
  - have a very limited role in the management of acute musculoskeletal conditions.
  - often used to treat musculoskeletal pain due to concerns about paracetamol efficacy and NSAID adverse reactions, but only have modest benefit and significant harm.
- Patients at increased cardiovascular risk:
  - Avoid NSAIDs in patients with established cardiovascular disease e.g., heart failure or stroke, or at high risk of CV disease.

- If treatment with an NSAID is necessary for a patient with risk factors, consider the following:
    - Naproxen provides the least cardiovascular risk of all the NSAIDs, but has a higher risk of gastrointestinal adverse effects.
    - Low dose aspirin may reduce the increased cardiovascular risk associated with NSAIDs, but it increases the risk of gastrointestinal adverse effects.
- Patients at increased gastrointestinal risk:
    - Avoid NSAIDs in patients with active peptic ulcer disease or gastrointestinal bleeding.
    - If treatment with an NSAID is necessary for a patient with risk factors, consider the following:
        - Diclofenac has a lower risk of gastrointestinal adverse effects due to its short half-life, but has high cardiovascular risk.
        - A proton pump inhibitor for prophylaxis.

- Oral NSAIDs (first line therapy)

Oral NSAIDs

- About 60% of patients will respond to any NSAID, those who do not respond to one may respond to another.
- There is no rationale for using more than one NSAID at a time (excluding low dose aspirin).
- Avoid in active peptic ulcer disease, high bleeding risk, established cardiovascular disease.
- Use the minimum effective dose of NSAID for the shortest time possible.
- Avoid continuing NSAIDs if there is no benefit or treatment is harmful.
- Consider topical NSAIDs to reduce the need for oral NSAIDs.
- Supply either:
    - Diclofenac 25 - 50 mg two or three times a day. Maximum daily dose of 200 mg, or
    - Ibuprofen 200 - 400 mg three to four times a day. Maximum daily dose of 2400 mg, or
    - Mefenamic acid 500 mg three times a day. Maximum daily dose of 1500 mg, or
    - Naproxen sodium 275 - 550 mg twice a day. Maximum daily dose of 1375 mg.

      Note: 275 mg of naproxen sodium = 250 mg naproxen.

- Oral Paracetamol

Oral Paracetamol

- Consider trialling paracetamol if NSAIDs are contraindicated or not tolerated.
- Evidence indicates that paracetamol is ineffective for nonspecific low back pain. However, some patients may find benefit.
- Supply paracetamol, either:
    - 1 g orally, every 4 to 6 hours as required. Maximum dose of 4 g in 24 hours, or
    - Controlled release 1.33 g orally, every 8 hours as required.

- Topical NSAIDs

Topical NSAIDs

- Ibuprofen: Rub 4 -10 cm into the affected area if needed, up to 4 times daily.

- Diclofenac:
  - 1% gel: rub into the affected area 3 or 4 times daily.
  - 2% gel: rub into the affected area twice daily.
- Piroxicam: Apply to the affected area 3 or 4 times daily.

Referral

Determine patient's regular general practitioner or practice and arrange appropriate referral:

- Immediate emergency referral:
  - Risk factors for spinal infection: fever, recent bacterial infection e.g., urinary tract infection, IV drug abuse, or immune suppression (from corticosteroids, transplant or HIV, or recent epidural or spinal anaesthesia.
  - Severe pain e.g., 9 - 10/10 on pain scale.
  - Severe or progressive neurological deficit or muscle weakness.
  - Acute onset of bowel or bladder dysfunction.
  - Major trauma e.g., vehicle accident or fall from height.
  - Risk of spinal fracture following recent significant trauma, or minimal trauma with suspected vertebral compression fracture.
  - History of cancer, and recent history of back pain at night or unexplained weight loss.
- Immediate general practitioner referral:
  - Young or older people (aged < 20 years and > 55 years).
  - Numbness.
  - Lower back pain that is:
    - at multiple sites
    - worse when lying on back
    - of sudden onset
    - causing any disability not already listed in emergency referral
    - radiating away from lower back area
- 1 week general practitioner referral:
  - Worse after sitting down for long periods.
  - Pain that is persistent and progressively worsening, in particular > 4 weeks.
- 4 to 5 week general practitioner referral:
  - Age < 20 years or > 55 years.
  - Younger person with:
    - morning stiffness
    - improvement with exercise
    - alternating buttock pain
    - awakening due to back pain during the second part of the night

Documentation and general practitioner feedback

- Ensure that the patient:
  - has read and signed the Patient Information and Informed Consent Form (to be kept at the pharmacy)
  - has completed the EURO-QOL EQ-5D questionnaire (hard copy to be kept at the pharmacy)
  - is informed of follow-up phone call conducted by the research team
- Provide date and time to patient of phone call (14 days after consultation in the pharmacy)
- Document the consultation by recording relevant information of the consultation on the data collection spreadsheet (for research purposes)
- Provide an electronic summary to the patient's regular general practitioner.

Provide an electronic summary to the patient's regular general practitioner.

- For all patients, provide a summary of the consultation, including non-prescription medicines provided.
- If referral was provided, indicate the criteria for referral.

Visit Health Link connect portal

Information

Clinical resources

- NSW Therapeutic Assessment Group - Low back pain
- ACI - Acute low back pain
- Low Back Pain. In: eTG complete [Internet]. Melbourne: Therapeutic Guidelines Limited; 2017 Mar.
- Australian Medicines Handbook 2017 (online). Adelaide: Australian Medicines Handbook Pty Ltd; 2017 Jan.
- National Institute for Health and Clinical Excellence (2017) Low back pain and sciatica overview. NICE guideline
- Benrimoj SI. A Cost-Benefit Analysis of Pharmacist Only (S3) and Pharmacy Medicines (S2) and Risk-Based Evaluation of the Standards. 2005.
- Rutter P, Newby D. Community pharmacy: symptoms, diagnosis and treatment. 2016; 3e

Patient information

- Pharmacy Guild of Australia - Pain diary
- Better health channel – Back pain – disc problems
- Health direct – Back pain prevention

Sources

References

1. Downie A, Williams C, Henschke N, Hancock M, Ostelo R, De Vet H, et al. Red flags to screen for malignancy and fracture in patients with low back pain: Systematic review. BMJ (Online). 2013;347.

Page Information

Information about this HealthPathways document (420331):
Document Owner: Not assigned (see Who's Who)
Last Updated:
Next Review:
Keywords:

Have you read the disclaimer?

© 2008-2018 WentWest Ltd

No content in this website may be reproduced or transmitted in any form or by any means, electronic, mechanical, recording, or otherwise, without the prior written approval of WentWest Ltd, and Canterbury District Health Board (where the original source of the content was from Canterbury District Health Board). For information on obtaining permission for reproduction or transmission of the content please contact info@healthpathways.org.nz.

HealthPathways

For Pharmacists- Period pain Pharmacy Protocol

This protocol is for community pharmacists participating in the trial of a collaborative minor ailments service. The protocol offers best practice advice on the care of adults aged ≥ 18 years with symptoms suggestive of period pain (primary dysmenorrhoea).

Disclaimer

About

About period pain

About period pain (primary dysmenorrhoea)

- A typical presentation involves lower abdominal cramping pains shortly before (6 hours) and for 2 hours, or possibly 3 days after, the onset of bleeding.
- May also involve back pain, nausea and / or vomiting in up to 50% of patients.
- 50-90% of women report experiencing some symptoms.
- Primary dysmenorrhoea is menstrual pain without organic pathology:
  - It is most common in adolescents and women in their early twenties.
  - Associated with late or early menarche, prolonged and heavier than normal menstrual flow, low bodyweight and body mass index, inadequate physical exercise, genetic predisposition, active and passive cigarette smoking, low socioeconomic status, diet, stress, and mental illness.[1]
- Secondary dysmenorrhoea has the same clinical features as primary dysmenorrhoea:
  - It occurs in women with conditions including pelvic inflammatory disease, endometriosis, adenomyosis, or uterine fibroids.
  - Usually affects women many years after menarche, typically aged > 30 years.

Red Flags

Send to emergency department immediately if vaginal bleeding is

- present with new onset severe pelvic pain.
- unusually heavy with lots of clots.

Consultation

1. Ask patient
   - their age
   - about pain

Pain

- Nature of pain: A great deal of overlap exists between primary and secondary dysmenorrhoea.
- Severity of pain:
  - Pain is not usually severe in primary dysmenorrhoea, the severity decreasing with the onset of menses.
  - Ask about severity level, using one of the following:
    - Numerical rating scale from 0 (no pain) to 10 (worst pain)
    - Visual analogue scale e.g., 10cm unmarked line with "no pain" at the left end and "worst imaginable pain" at the right end.
    - Words to describe pain e.g., none, mild, moderate, severe, or worst possible pain.

2. Identify any concerning features and refer appropriately, to general practitioner as below.

Concerning features

- Heavy or unexplained vaginal bleeding including bleeding at unexpected times in the cycle or in post-menopausal women
- Pelvic pain not around time of menses or chronic / ongoing pelvic pain
- Associated lower back and/or thigh pain
- Associated bladder and/or bowel symptoms
- Associated and disruptive emotional symptoms
- Change in vaginal discharge
- Pain with intercourse
- Signs of systemic infection
- New or worsening symptoms, especially in patients aged > 50 years

3. Consider causes of secondary dysmenorrhoea or other diagnoses.

Causes of secondary dysmenorrhoea or other diagnoses

- Endometriosis
  - Period pain and sometimes ovulation pain as well
  - Bowel and bladder symptoms
  - More likely to have pain in the lower back and thighs
  - Pain with sex
- Pelvic inflammatory disease
  - Does not always cause symptoms
  - Pelvic pain with menstrual disturbance
  - Deep pain during sexual intercourse
  - Change in vaginal discharge
  - Fever
- Ovarian cysts
  - Often asymptomatic
  - Pelvic pain with unusual vaginal bleeding
  - Pain during sexual intercourse
- Uterine cancer
  - Post-menopausal
  - Abnormal vaginal bleeding and discharge
  - Often no pain
- Premenstrual syndrome
  - Collection of physical and emotional symptoms
  - No underlying medical condition
  - Cyclic in nature
  - Tends to resolve on menstruation
  - Rarely seen prior to ovulation

Treatment

1. Provide non-pharmacological support.

Non-pharmacological support

If symptoms are considered to be a minor ailment condition, proceed with a standardised management approach.

Provide each patient a PSA Self-Care Fact Card.

Advise patient to:

- Apply local heat: e.g., with a hot water bottle or heat pack to lower abdomen. Heat is better than paracetamol for cramping/ tightness.
- Consider:

- high frequency TENS, which studies show is more effective for pain relief than placebo.[3]
- behavioural interventions, for which there is some evidence.[3]
- acupuncture, insufficient evidence.[3]

2. Consider:
    - pharmacological options, with an awareness of specific considerations.

Specific considerations for use of oral NSAIDs

- Contraindications: Pre-existing ulcer
- Precautions: Heart failure
- Drug interactions: Warfarin
- Impaired renal function: Lithium, digoxin, ACE inhibitors, diuretics

NSAIDs

- NSAIDs relieve primary dysmenorrhoea by suppressing prostaglandins in menstrual fluid.
- Ideally given 48 hours before menstruation is expected, or with onset of pain. Active within 3-60 minutes following oral administration.
- Advise patient to continue treatment for the first 48 to 72 hours of menses.

Dispense either:

- Ibuprofen 200 mg to 400 mg (1 - 2 tablets) 3 to 4 times daily. Maximum daily dose 1600 mg, or
- Mefenamic acid 500 mg (2 capsules) 3 times daily with food, or
- Naproxen sodium 550 mg (2 tablets) initially, then 275 (1 tablet) mg every 6 to 8 hours. Maximum daily dose 1375 mg. Note: 550 mg naproxen sodium is equivalent to 500mg naproxen.

    - complementary therapies

Complementary therapies

There is some evidence for Magnesium and Vitamin B1 (thiamine).[3] Other therapies have limited evidence.

- It is currently unclear what dose or regimen of magnesium treatment should be used.
- Vitamin B1 is shown to be an effective treatment for dysmenorrhoea taken at 100 mg daily.

Referral

Arrange referral if symptoms are not considered to be a minor ailment condition or patient is presenting with red flags or concerning features. Determine patient's regular general practitioner or practice and arrange appropriate referral:

- Immediate emergency department referral if vaginal bleeding is:
    - present with new onset severe pelvic pain
    - unusually heavy with lots of clots
- Immediate general practitioner referral:
    - Heavy or unexplained bleeding
    - Signs of systemic infection
- 2 - 3 week general practitioner referral:
    - Vaginal bleeding in post-menopausal women
    - Changes in vaginal bleeding
    - Pelvic pain not around time of menses or chronic / ongoing pelvic pain
    - Associated:

- lower back or thigh pain
- bladder or bowel symptoms
- disruptive emotional symptoms

- Change in vaginal discharge
- Pain with intercourse

Documentation and general practitioner feedback

- Ensure that the patient:
  - has read and signed the Patient Information and Informed Consent Form (to be kept at the pharmacy)
  - has completed the EURO-QOL EQ-5D questionnaire (hard copy to be kept at the pharmacy)
  - is informed of follow-up phone call conducted by the research team
- Provide date and time to patient of phone call (14 days after consultation in the pharmacy)
- Document the consultation by recording relevant information of the consultation on the data collection spreadsheet (for research purposes)
- Provide an electronic summary to the patient's regular general practitioner.

Provide an electronic summary to the patient's regular general practitioner.

- For all patients, provide a summary of the consultation, including non-prescription medicines provided.
- If referral was provided, indicate the criteria for referral.

Visit Health Link connect portal

Information

Patient information

Better health channel - Menstruation - pain (dysmenorrhoea)

Clinical resources

- Benrimoj SI. A Cost-Benefit Analysis of Pharmacist Only (S3) and Pharmacy Medicines (S2) and Risk-Based Evaluation of the Standards. 2005.
- Rutter P, Newby D. Community pharmacy: symptoms, diagnosis and treatment. 2016; 3e
- eTG Dysmenorrhoea. Mar 2014. (eTG July 2017 edition)
- Menstrual Disorders. In: eTG complete [Internet]. Melbourne: Therapeutic Guidelines Limited; 2014 Mar.
- Australian Medicines Handbook 2017 (online). Adelaide: Australian Medicines Handbook Pty Ltd; 2017 Jan.

Sources

References

1. Grandi G, Ferrari S, Xholli A, Cannoletta M, Palma F, Romani C, et al. Prevalence of menstrual pain in young women: What is dysmenorrhea? Journal of Pain Research. 2012;5:169-174.
2. Proctor ML, Murphy Pa. Herbal and dietary therapies for primary and secondary dysmenorrhoea. Cochrane database of systematic reviews (Online). 2001;(3):CD002124.

Select bibliography

- Akin M, Price W, Rodriguez G, Smith RP. Continuous low level heat wrap therapy as compared to acetaminophen for primary dysmenorrhoea. J. Reprod. Med. Obstet. Gynecol. 2004;49(9):739-745.

- Proctor ML, Smith CA, Farquhar CM, Stones RW. Transcutaneous electrical nerve stimulation and acupuncture for primary dysmenorrhoea. Cochrane Database Syst Rev. 2002;(1):CD002123.
- Proctor ML, Murphy PA, Pattison HM, Suckling J, Farquhar CM. Behavioural interventions for primary and secondary dysmenorrhoea [Systematic Review]. Cochrane Database of Systematic Reviews. 2007;(4).

Page Information

Information about this HealthPathways document (420803):

Document Owner: Not assigned (see Who's Who)

Last Updated:

Next Review:

Keywords:

Have you read the disclaimer?

© 2008-2018 WentWest Ltd

No content in this website may be reproduced or transmitted in any form or by any means, electronic, mechanical, recording, or otherwise, without the prior written approval of WentWest Ltd, and Canterbury District Health Board (where the original source of the content was from Canterbury District Health Board). For information on obtaining permission for reproduction or transmission of the content please contact info@healthpathways.org.nz.
